# Effects of phase synchronization and frequency specificity in the encoding of conditioned fear–a web-based fear conditioning study

**DOI:** 10.1371/journal.pone.0281644

**Published:** 2023-03-03

**Authors:** Elena Plog, Martin I. Antov, Philipp Bierwirth, Ursula Stockhorst

**Affiliations:** Experimental Psychology II and Biological Psychology, Institute of Psychology, University of Osnabrück, Osnabrück, Germany; La Sapienza University of Rome, ITALY

## Abstract

Oscillatory synchronization in the theta-frequency band was found to play a causal role in binding information of different modalities in declarative memory. Moreover, there is first evidence from a laboratory study that theta-synchronized (vs. asynchronized) multimodal input in a classical fear conditioning paradigm resulted in better discrimination of a threat-associated stimulus when compared to perceptually similar stimuli never associated with the aversive unconditioned stimulus (US). Effects manifested in affective ratings and ratings of contingency knowledge. However, theta-specificity was not addressed so far. Thus, in the present pre-registered web-based fear conditioning study, we compared synchronized (vs. asynchronized) input in a theta-frequency band vs. the same synchronization manipulation in a delta frequency. Based on our previous laboratory design, five visual gratings of different orientations (25°, 35°, 45°, 55°, 65°) served as conditioned stimuli (CS) with only one (CS+) paired with the auditory aversive US. Both CS and US were luminance or amplitude modulated, respectively, in a theta (4 Hz) or delta (1.7 Hz) frequency. In both frequencies, CS-US pairings were presented either in-phase (0° phase lag) or out-of-phase (90°, 180°, 270°), resulting in four independent groups (each group *N* = 40). Phase synchronization augmented the discrimination of CSs in CS-US contingency knowledge but did not affect valence and arousal ratings. Interestingly, this effect occurred independent of frequency. In sum, the current study proves the ability to successfully conduct complex generalization fear conditioning in an online setting. Based on this prerequisite, our data supports a causal role of phase synchronization in the declarative CS-US associations for low frequencies rather than in the specific theta-frequency band.

## Introduction

Phase synchronization in the theta band is regarded as an important mechanism for synaptic plasticity and communication between and within brain regions [1, 2]. The assumption is mainly based on work in rodents [e.g., 3, 4] and human EEG-studies [5, 6], showing that theta-phase synchronization increases during encoding and successful retrieval of memory content. Most human studies that examined the role of theta-phase synchronization in memory are correlative in nature with (theta) synchronization as an “epiphenomenal oscillatory signature of memory” [1, p. 1]. Recently, Clouter et al. [7] provided first experimental evidence of a *causal* role of phase synchronization for the formation of episodic associative memory in humans using a simple but elegant non-invasive technique. They applied repetitive rhythmic sensory stimulation in the theta band and concurrently presented visual and auditory stimuli. The theta modulation of stimulus features allows experimental control over phase synchrony of the input. Intriguingly, phase-synchronized compared with asynchronized presentation resulted in an improved memory recall of video-tone pairs [7]. Moreover, this synchronization effect was specific for the theta frequency, whereas it did not occur at an alpha (10.5 Hz) or a delta (1.7 Hz) stimulation frequency. The findings were later replicated and extended [8], making a strong case that phase synchronization in the theta band is causally involved in the formation of multimodal declarative memory traces.

In terms of memory systems, classical fear conditioning is typically considered a separate, non-declarative type of memory with different neural correlates rooted in the brain’s defensive system [9–11]. Yet, classical conditioning typically also relies on multimodal associations between a neutral stimulus (conditioned stimulus, CS) in one modality (e.g., a visual stimulus) and an aversive, unconditioned stimulus (US) processed by a different sensory system (e.g., the nociceptive in case of an electric shock, or the auditory in case of an aversive tone). It is well established that during fear conditioning, the sensory information from the CS and US converges in the lateral amygdala [LA, 12–14]. Here, activating the weaker CS synapses and strong US synapses in close temporal proximity is crucial to initiate a strengthening of the weak CS synapse, enabling the CS to elicit a fear response by itself [9, 12]. Common oscillatory mechanisms (including synchronization in the theta band) may be shared across different memory systems of the brain [15]. While various studies in animals and humans show the importance of synchronization in the theta band during different stages of fear acquisition and extinction [16–19], its causal role in forming CS-US associations was unknown. To close this gap and focus on the causal role of theta synchronization in fear conditioning, we extended earlier findings in declarative memory [7, 8], applying repetitive rhythmic sensory stimulation to classical fear conditioning in humans [20]. We investigated the effects of theta-phase synchronized vs. asynchronized CS-US input on fear acquisition in a CS-generalization paradigm. In a 2-day lab-based fear conditioning paradigm, we modulated the luminance of five visual CSs and the amplitude of the aversive auditory US sinusoidally at 4 Hz. During acquisition, we then presented the overlapping CS+US in two independent groups of participants either with a phase shift of 0° (i.e., synchronized) or with a phase lag of 90°, 180°, and 270° (i.e., asynchronously). Intriguingly, the effects of theta-phase synchronization varied with different fear measures. Synchronized (as compared to asynchronized) presentation augmented contingency knowledge (US-expectancy) and affective evaluation, both assessed via ratings. However, it did not amplify conditioned responding in physiological arousal and visuocortical engagement. This suggests that the applied stimulation technique is better suited for declarative-like measures of a human (fear) conditioning task.

Although the previous studies [7, 8, 20] deliver initial evidence for phase synchronization as a shared mechanism across declarative and fear conditioning tasks, it remains to be examined if the phase-synchronization effect in fear conditioning is specific to the theta band. The current study was designed to address this question. In accordance with Clouter et al. [7], here, we examined frequency specificity by contrasting the effects of synchronization vs. asynchronization not only in the theta but also in the delta band (1.7 Hz). Although the slow delta-frequency band is also associated with memory processing, it predominantly occurs during slow-wave-sleep, where it is regarded as an important factor for memory consolidation [2, 21] which is not tested within the current study (i.e., we did not assess delayed recall).

An important feature of the present study is its online character: The Covid-19 pandemic forced researchers to adapt to new standards of contact restrictions and hygiene concepts. For that reason, our study is, as far as we know, one of the first to test a web-based fear conditioning paradigm [for other examples: 22, 23]. The choice of an online-format was especially suitable because our laboratory study revealed effects in the rating-based measures only, which are easily assessable online. Thus, we used repetitive presentation of the visual and auditory stimuli in 4 Hz (theta, identical to the laboratory study) and at 1.7 Hz (delta). To confirm our previous findings, our procedure was adopted with maximal similarity to the laboratory study [20].

Nevertheless, as a consequence of the previous findings as well as the web-based approach, we implemented a few adjustments: Since the effects of synchronization were restricted to the ratings on day 1 in the lab-based study, here we only used a 1-day web-based conditioning task (with habituation, fear acquisition, and extinction). Removing day 2 should not interfere with confirming our previous results. A second adjustment concerns the volume of the auditory US that should be aversive enough to elicit conditioned fear. In a web-based study we have no direct access to a participant’s hardware at home and cannot measure the actual sound pressure level. Thus, we decided to use an individually adjusted titration procedure to establish a sound volume that is unpleasant but individually tolerated. As classical fear conditioning is a passive task, we added a simple control task (between learning phases) to ensure that (a) participants have not reduced the audio volume, and (b) that participants are still in position in front of the computer screen.

Based on our previous findings [20] and the assumption that phase synchronization is frequency-specific to the theta frequency, we hypothesize that theta-phase synchronization (vs. asynchronization) improves the ability to discriminate between the CS+ and CS- gratings in valence, arousal, as well as US-expectancy ratings, i.e., it determines the width of the generalization across the CS orientations. Thus, for the theta frequency, we expect a narrower generalization (i.e., better discrimination between CS+ and neighboring orientations) after phase synchronization as compared to a broader generalization (i.e., attenuated discrimination between CS+ and most similar CS- gratings) after asynchronous CS-US presentation (orientation x synchronization interaction for customized contrast fits). In contrast, for the delta frequency, we expect a broad generalization for both, in-phase and out-of-phase groups.

In sum, the present study aims at extending the initial knowledge of synchronization in Pavlovian conditioning by examining whether the memory-improving effect is specific for theta-band stimulation and does not occur in the delta band. Due to the Covid-19 pandemic, we transferred our complex fear conditioning paradigm into a web-based procedure. Thus, the study also aims at providing knowledge of how to implement, control and validate a complex conditioning task in a web-environment. Our results prove a successful implementation of a complex generalization fear conditioning protocol in a web-based approach that is sensitive to fear acquisition and extinction. However, synchronization affected CS-US contingency knowledge in both theta and delta frequency, suggesting that low frequency (theta and delta) rather than theta-specific entrainment supports the (predominantly declarative) memory of CS-US contingency.

## Materials and methods

### Preregistration

The study was submitted to OSF as “Plog, E., Antov, M. I., Bierwirth, P., & Stockhorst, U. (2021, September 12). Effects of phase synchronization and frequency specificity in the encoding of fear—an online study. The preregistration is publicly available at osf.io/bgq9z”. For the dataset including raw and z-transformed rating values see **S1 Dataset**.

### Participants

Based on our laboratory experiment, the sample size was determined, using *Superpower* in the online shiny app (https://arcstats.io/shiny/anova-exact/ and https://arcstats.io/shiny/anova-power/) [24]. The algorithm uses Monte Carlo simulations to estimate power for an ANOVA. It allows power estimation for the specific form of an expected interaction, not merely for any kind of a significant interaction. Predicted effects are given by entering the means and standard deviations (SDs) [24]. To obtain a power of at least 80% with an alpha error of 0.05 for the hypothesized 5 x 2 x 2 interaction, we entered the means (*M*) and a common *SD* of the 5 (CS orientation) x 2 (theta-synchronization) interaction from our previous laboratory study [20] (see **S1 Table** for entered *M* and common *SD*s). For delta, we expected a broad generalization in both, in-phase and out-of-phase groups, similar to the theta out-of-phase group. For the delta-condition we therefore entered the *M* and *SD* from the theta out-of-phase group from our previous study [20] as an estimation for both, in-phase and out-of-phase effects (see **S1 Table**). The power analysis revealed a sample size of 160 participants, i.e., 40 participants in each of the four independent groups (see also section *Experimental design and stimuli*).

All participants were university students between 18 and 35 years. They were recruited via mailing lists of different universities and flyers on the campus of the University of Osnabrück. Female participants were only included if using monophasic oral contraceptives (pill) and were specifically instructed to attend our study between the 6^th^ and 21^st^ day of pill-intake. The screening for inclusion and exclusion criteria was conducted with an online questionnaire via SoSci-Survey (https://www.soscisurvey.de). Only participants that were identified as eligible by the screening received a link to continue to the main experiment hosted on Pavlovia (https://pavlovia.org/). Participants were excluded when suffering from acute or chronic physical and/or psychiatric disorders (e.g., migraine and epilepsy and neurological disorders). Further exclusion criteria encompassed impaired hearing, uncorrected vision deficits, tinnitus, acute medication (e.g., antibiotics, sedatives, antidepressants), drug abuse and an average alcohol consumption exceeding 20 g or 40 g ethanol per day (for women and men, respectively). Additionally, participants were screened for posttraumatic stress disorder (PTSD), using a translated version of the Posttraumatic Stress Diagnostic Scale [25, 26] and excluded if they met the DSM-IV criteria for PTSD. Moreover, technical inclusion criteria were demanded, comprising a laptop or desktop PC with an updated version of Windows 10 or macOS (10.12 or higher), participating via smartphone or tablet led to exclusion. Subjects had to use wired headphones connected to the laptop/PC to avoid possible time lags caused by wireless transmission. We also asked participants to use either Google Chrome, Edge (Windows 10 users), or Safari browsers (Apple users), as those delivered the best timing in our pretests.

Overall, 346 participants started the online screening. Of those, 10 discontinued before finishing the screening, and 54 were excluded due to ineligibility (e.g. migraine, epilepsy, substance abuse) and never started the main experiment. After passing the screening, participants were accepted in consecutive order to start the main experiment. Of those, 38 participants discontinued the study before finishing the main experiment and were excluded from analysis. Additionally, seven participants were excluded due to a technical error.

We balanced the four experimental groups in terms of an equal number of men/women per group, and the trial order (equal number of participants with list A and list B per group). To achieve this, the online data collection assigned each participant that passed the screening to one of 16 subgroups (4 experimental groups x 2 sexes x 2 trial order lists). We needed complete datasets (finished experiment) from 160 participants, that also passed the cut-off criterion of the compliance control task. The check of the compliance criterion, however, was done offline by our team. Moreover, in an online experiment, multiple participants can participate simultaneously. Therefore, we unwillingly collected data from more than 160 participants. Of the 237 participants that finished the full experiment, 55 missed the 50% compliance criterion, and were excluded, leaving a sample of *N* = 182. The software delivers precise time stamps for each participant. As the exact time of participation can be assumed to be independent of any study-related variables, we used these time stamps to exclude those participants that were collected beyond the planned *N* = 160. Chronologically, the last participants in each group exceeding the planned sample size were excluded (*N* = 20 in total; 12 women). Importantly, these final exclusions were based solely on the time stamps provided by the software (i.e., blind to any behavioral data). Thus, the final sample consisted of 80 men, 80 women and 2 non-binary participants (age: *M* = 23.49, *SD* = 3.16). As the number of non-binary participants was insufficient to attend each of the 4 independent groups, the reported data analyses will only include male and female (N = 160) participants.

The study was approved by the ethics committee of the University of Osnabrück and conducted in accordance with the Declaration of Helsinki guidelines. Written informed consent was obtained from all participants after their confirmation of full understanding of the procedure. Participants that finished screening and the conditioning procedure received a voucher over 15 EUR. Students of the university of Osnabrück were free to choose between the voucher or 1.5 course credits.

### Experimental design and stimuli

The study followed a 5 x 2 x 2 mixed factorial design (per learning phase, see *Conditioning procedure*), with 5 CS as the within-subject factor *orientation*, and the between-subjects factors *synchronization* (in-phase vs. out-of-phase), and *frequency* (theta [4 Hz] vs. delta [1.7 Hz]). Thus, the design had four independent groups *theta (in-phase)*, *theta (out-of-phase)*, *delta (in-phase)*, and *delta (out-of-phase)*.

**Fig 1:**
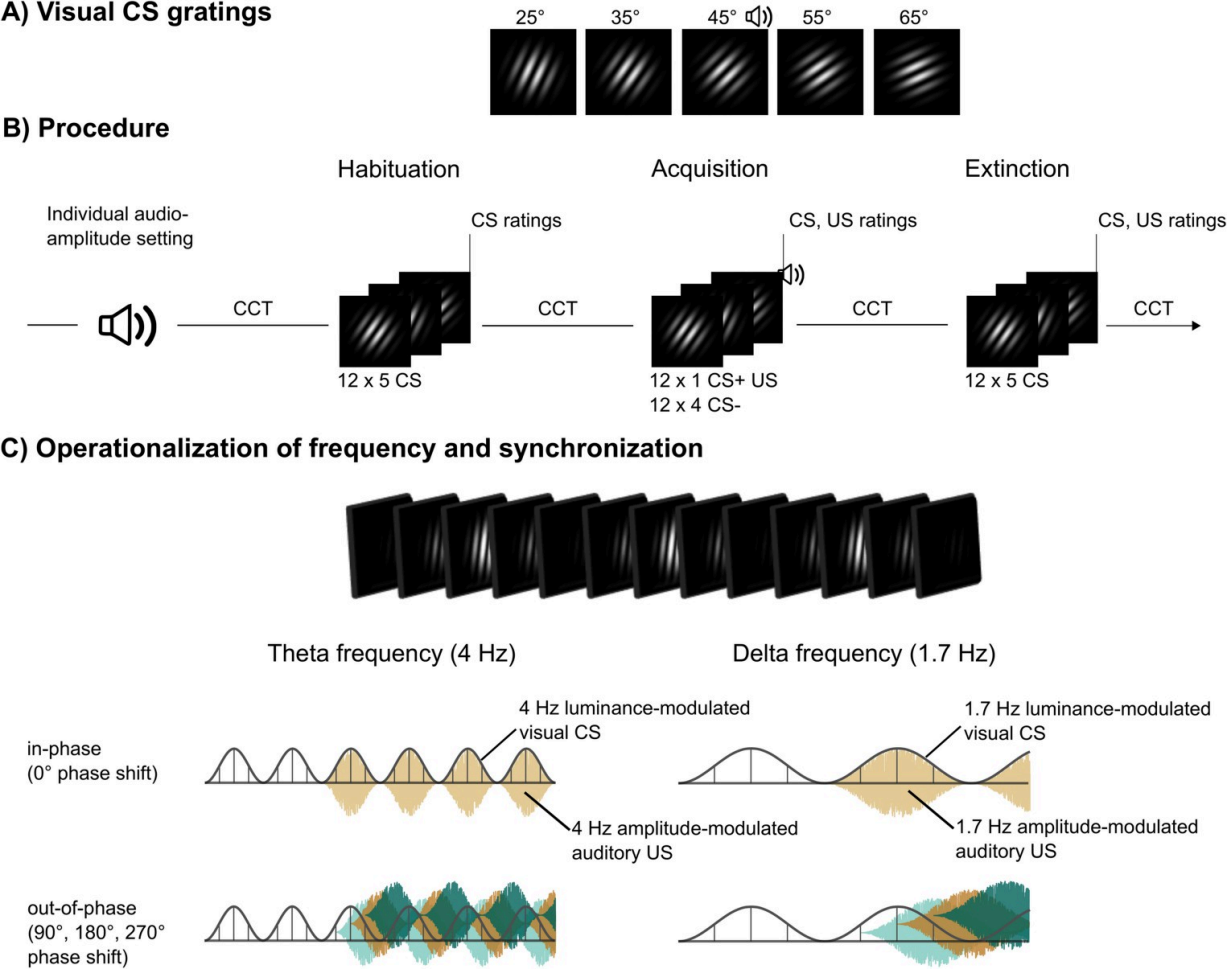
Experimental design. Gabor gratings, conditioning procedure, and the operationalization of in-phase versus the out-of-phase in a theta- (4 Hz) or delta- (1.7 Hz) frequency band. **(A)** For CS, we used Gabor gratings that only differed in orientation by 10°. The 45° orientation served as CS+ and was therefore paired with the US during acquisition. The other four orientations (25°, 35°, 55°, and 65°) were used as CS- gratings, hence never paired with the US. **(B)** The fear conditioning paradigm comprised the learning phases habituation, acquisition, and extinction. Prior to habituation, participants conducted the individual audio-volume setting (titration) to select an US intensity that is extremely unpleasant but not painful. Within a learning phase, each CS orientation was presented 12 times. Only during acquisition, the US with the individually set intensity was presented together with the CS+ (45°) orientation. After each learning phase, valence and arousal ratings were conducted for the CS and–after acquisition–the aversive US. US-expectancies were rated after acquisition and extinction. At four time points (before and after habituation, after acquisition, and after extinction) participants conducted the unheralded compliance control task (CCT). **(C)** Operationalization of the in-phase (0° phase shift) versus out-of-phase (90°, 180°, 270° phase shift) synchronization in a theta- (4 Hz) or delta- (1.7 Hz) frequency band. Each visual CS and auditory US was sinusoidally luminance or amplitude modulated, respectively, at either 4 Hz (theta) or 1.7 Hz (delta). The left column shows phase shifts for the theta band: in-phase, i.e., 0° (beige) shift at the top and out-of-phase, i.e., 90° (light green), 180° (brown), 270° (dark green) shift at the bottom. The right column depicts the same phase-shifts for delta.

As visual CS we used five high-contrast, black-and-white Gabor gratings (i.e., sine-wave gratings with a Gaussian envelope, **Fig 1A**) with a low spatial frequency. The five visual CS differed only in orientation (25°, 35°, 45°, 55°, 65°) [20]. Each grating was presented in the middle of the screen on a dark grey background for 5 s (habituation and extinction) or 7 s (acquisition). The auditory US was the same 2 s, broadband white noise (20 Hz– 22 kHz, 44100 Bit/s, 16 Bits/sample) used in our previous laboratory study [20]. While US intensity was constant (max. 96.5 dB[A]) in the laboratory study, we now included an individual titration procedure at the beginning of the online study allowing each participant to set up a highly aversive yet tolerable volume of the US (see *Overall Procedure*).

The visual CS and the auditory US were modulated at either 4 Hz (theta group) or 1.7 Hz (delta group). The visual CS were luminance modulated from 0–100% luminance, the auditory US was amplitude modulated (0–100%) by multiplying the signal vector with a 4 Hz or 1.7 Hz sine wave, respectively.

The auditory white noise was downloaded from random.org (https://www.random.org/audio-noise/) and subsequently amplitude modulated (4 and 1.7 Hz) using a custom Matlab script (version R2021a). In the previous laboratory study [20], the luminance of the gratings was modulated on a frame-by-frame basis. However, the feature to generate grating stimuli in PsychoPy online, was not supported by the time we programmed the experiment. Thus, we wrote an offline python script that created the desired Gabor-Gratings, modulated their luminance with a 1.7 or 4 Hz sine-wave, and created (5–7 s long) video clips to upload and use as CS online. The timing of the auditory US (theta- or delta-modulated audio file) was defined in relation to the start of the video clip at the beginning of a trial. The experimental procedure was created in PsychoPy [27] (version v2021.2.3), uploaded as PsychoJS (java-script code) and hosted by the online platform Pavlovia (http://pavlovia.org).

### Conditioning procedure

The study comprised the learning phases habituation, fear acquisition, and extinction (**Fig 1B**). Within each learning phase, the 5 CS orientations were presented 12 times each, resulting in a total of 60 CS presentations per learning phase [20]. The auditory US was presented during fear acquisition only. During inter-trial-intervals (ITIs), a white fixation cross, was presented in the center of the screen with intervals varying randomly between 4.5 s and 6.5 s. The duration of each CS differed between learning phases: During habituation and extinction, each CS was presented for 5 s. Within acquisition, the 7-s long, 45° CS grating (CS+) co-terminated with the aversive auditory US in the last 2 s of CS presentation. To ensure perceptual comparability between the gratings, the duration of all 5 CS gratings (CS+ and CS-) in acquisition were extended by 2 s, leading to a 7-s duration. As in the previous laboratory study, we selected the 45° orientation as CS+ for all subjects to provide the intended generalization design with symmetrically distributed CS- gratings around the CS+. Previous studies did not reveal systematic differences between CS orientations prior to acquisition and also showed successful conditioning across orientations [28, 29].

In all four experimental groups, the 2-s US overlapped with the last 2 s of the 7 s visual CS+ during acquisition. Participants in the theta and delta *in-phase groups* received 12 trials of CS+ US pairing where the oscillating visual CS+ and auditory US had a 0° phase shift. Participants in the theta and delta *out-of-phase groups* also received 12 trials of CS+ US pairings. However, for these 12 trials, each participant in the out-of-phase group received 4 CS+ US pairings with a phase-shift of 90°, four trials with a phase shift of 180°, and 4 trials with a phase-shift of 270° (pseudorandom order). In all 4 groups, we also accounted for the fact, that the transduction of auditory signals is faster compared to visual signals. Therefore, we added a fixed lag of 40 ms to the onset of the auditory US (for details, see [7, 8, 20]).

The sequence of the five CS gratings in each learning phase followed one of two trial orders that were counterbalanced within groups. These orders (trial lists) were created in a pseudorandomized way, with the only restriction of allowing no more than two consecutive gratings of the same orientation. Within acquisition, both trial lists started with a so-called booster session, i.e., a CS+US pairing occurred in five of the first seven trials [cf. 20, 28, 30].

### Dependent variables

Due to the online restrictions and to replicate the main findings of the laboratory study [20], we assessed US-expectancy, valence, and arousal ratings, but not physiological arousal (measured via skin conductance responses) and visuocortical engagement (measured via steady-state visually evoked potentials). We adapted the 9-point Self-Assessment Manikin [SAM, 31] to an online version within PsychoPy to assess the valence and arousal ratings.

After habituation, acquisition, and extinction, participants were asked to evaluate each of the differently oriented gratings for its valence (from unpleasant 1 to pleasant 9) and arousal (from calm 1 to arousing 9). In addition, US-expectancy was assessed after acquisition and after extinction: Participants were asked to rate the likelihood that a US will follow each of the 5 CS gratings on a scale from -5 (certainly no US) over 0 (uncertain) to 5 (certainly US).

### Overall procedure

The study consisted of two consecutive parts: 1) a screening and 2) the conditioning session. As described in the section *Conditioning procedure*, conditioning comprised the learning phases habituation, acquisition, and extinction that were separated by the valence, arousal, and US-expectancy ratings, resting periods, as well as the compliance control tasks (**Fig 1B**). Each learning phase took 10 to 13 minutes, depending on the ITI (between 4.5 and 6.5 s) and the duration of CS stimuli that was extended for acquisition.

### Screening

The screening (presented via https://www.soscisurvey.de/) included a description of the general procedure (participant information), checked the relevant inclusion and exclusion criteria (e.g., physical and psychological health, technical requirements, via self-report questionnaires), and obtained informed consent. At the end of the screening, eligible participants underwent the individual titration of US intensity (described below). Participants were instructed to set their laptop/desktop PC audio to the maximum volume (100%) and were automatically redirected to the conditioning procedure. The average duration for completing the screening and conditioning part was 1 hour and 5 minutes (*SD* = 32 minutes).

### US-intensity titration

Prior to the habituation phase, participants were instructed to individually adjust the volume of a test stimulus (a low-amplitude, frequency-modulated white noise US, same frequency composition as the final US) to a level that is aversive but not painful, using a clickable controller. In a second step, the previously adjusted level was rated on a 10-point Likert scale from 0 (not unpleasant at all) to 10 (extremely unpleasant). A rating of 7 or higher finished the evaluation and saved the volume setting for the US. If the tone unpleasantness was rated less than 7, the volume was increased in small steps until it reached an unpleasantness rating of 7 or higher (*M* = 8.52).

### Instructions

In the beginning of each learning phase, participants were instructed to sit comfortably and avoid any movement (except blinking) for the duration of the stimulus presentation. We also kindly instructed them to dim the room, if possible, to provide the best vision of the dark gratings. Participants were informed that a fixation cross will be presented in the center of the screen, followed by a frequency-modulated black and white grating that differed in orientation. In the resting periods between the learning phases, participants were encouraged to relax their eyes, without leaving the position in front of their laptop/PC. Before acquisition, we instructed the participants that during the next phase, a loud, pulsating noise will follow one of the gratings, without specifying which of the five gratings. Prior to extinction, we did not specifically inform them that the US will never follow the CS+ anymore.

### Compliance control task

Compliance control was conducted to evaluate the participants’ visual and auditory perception and thus their attention towards the experiment. At four time points throughout the experiment, a random number (between 1 and 4) of low volume auditory beeps were presented monoaurally either to the left or the right ear. After the presentation, participants were asked to identify (instructions on the screen) 1) how many beeps they had just heard and 2) on which ear they had received the tone (left or right ear). The compliance control task had several aims: 1) Playing the beep sounds at a low-volume (1/4 of the previously chosen “aversive” setting) provided a control for an adequate US-intensity titration of each participant. If participants muted their audio, chose a low volume at the individual setting in the beginning, or lowered their device volume during the task, they would be unable to hear the beep at all, and miss our criteria for successful participation. 2) Playing the beeps sounds without prior notice and presenting the questions (concerning number of right or left direction) written on the screen ensured that participants kept sitting in front of the screen during the experiment. 3) The use of monaural beeps ensured that participants wear headphones, as instructed, and additionally, wear them correctly on/in both ears. Our a priori-defined compliance criterion allowed a maximum of 2 out of 4 errors in both, the question about the number of presented beeps as well as the question about the side of beep occurrence (i.e., a minimum of 50% in each of the two questions). The distribution of errors is listed in **Table 1**.

**Table 1:** Distribution of errors in the compliance control task^1^.

Errors in *Number of beeps*	Participants	Errors in *Monoaural presentation side*	Participants
0	132	0	2
1	30	1	156
2	35	2	22
3	18	3	36
4	2	4	1

^1^Of note, error distributions were conducted including the two non-binary participants.

### Realizing the online set up

As described earlier, for the screening session we used the online platform SoSciSurvey. The main part of the study was programmed in Psychopy [27] and hosted by Pavlovia (http://pavlovia.org). We received payed support from the consultancy team of PsychoPy (https://psychopy.org/consultancy.html) on some specific programming issues. In order to anonymously identify each participant between both platforms, a pseudonymized ID was generated during the initial screening part in SoSciSurvey. In case of eligibility, participants were redirected to the main study with a URL that included the pseudonymized ID. To randomly assign each participant to one of the groups, we used the VESPR (Vertical Enhancement of Statistics and Psychological Research) study portal [32]. Due to a minor error that prevented the correct counterbalancing across the groups defined by synchronization, frequency, and trial lists, we switched to an assignment within PsychoPy for the last 69 participants of our sample. However, VESPR was used for all participants to guarantee the equal distribution of men and women. At the end of the experiment, the ID was displayed on the screen. Participants were instructed to send us an email, that included the ID and the selected compensation (voucher or course credits). After confirming the correctness of the ID, we compensated them with the voucher or course credits.

### Statistical analysis

For each outcome measure (US-expectancy ratings, valence and arousal ratings) and learning phase (habituation, acquisition, extinction) we performed a repeated-measures mixed ANOVA including the within-subject factor *orientation* (25°, 35°, 45°, 55°, 65°) and the between-subject-factors *synchronization* (in-phase vs. out-of-phase) and *frequency* (theta vs. delta). Of note, we will report Greenhouse-Geisser corrected values. The significance level was set to *p* < .05.

Before looking for effects of phase synchronization in the theta- or delta- frequency band, one major prerequisite concerns the ability to induce fear conditioning in a web-based study. To validate successful fear acquisition and extinction, we analyzed rating patterns across the CS orientation, independent of synchronization and frequency conditions. To account for the fear generalization paradigm with CS- gratings symmetrically distributed around the CS+ grating, we utilized customized contrast weights. Over all groups (i.e., independent of factors frequency and synchronization), we used generalization weights (-0.529, 0.247, 0.564, 0.247, -0.529) to validate the successful conditioning with the greatest increase towards the CS+ orientation. Additionally, we included the factor *learning phase (LP)* to examine a possible decrease in fear responses from acquisition to extinction, validating successful extinction.

To analyze the hypothesized effects within each measure, we first examined the orientation x synchronization x frequency interaction within the ANOVA described above. Due to unexpected pre-conditioning differences in ratings of valence and arousal with a linear decrease or increase (25° to 65°) with grating orientation, we additionally conducted habituation-corrections within each participant. For this, individual valence and arousal ratings given after acquisition and extinction were divided by the participant’s corresponding rating after habituation. The result was multiplied by 100, leading to a percentage score. The habituation-corrected valence and arousal ratings are additionally shown in result figures or supporting information for a better visualization of fear generalization patterns only. The statistics in the result section are nonetheless based on raw data.

**Fig 2:**
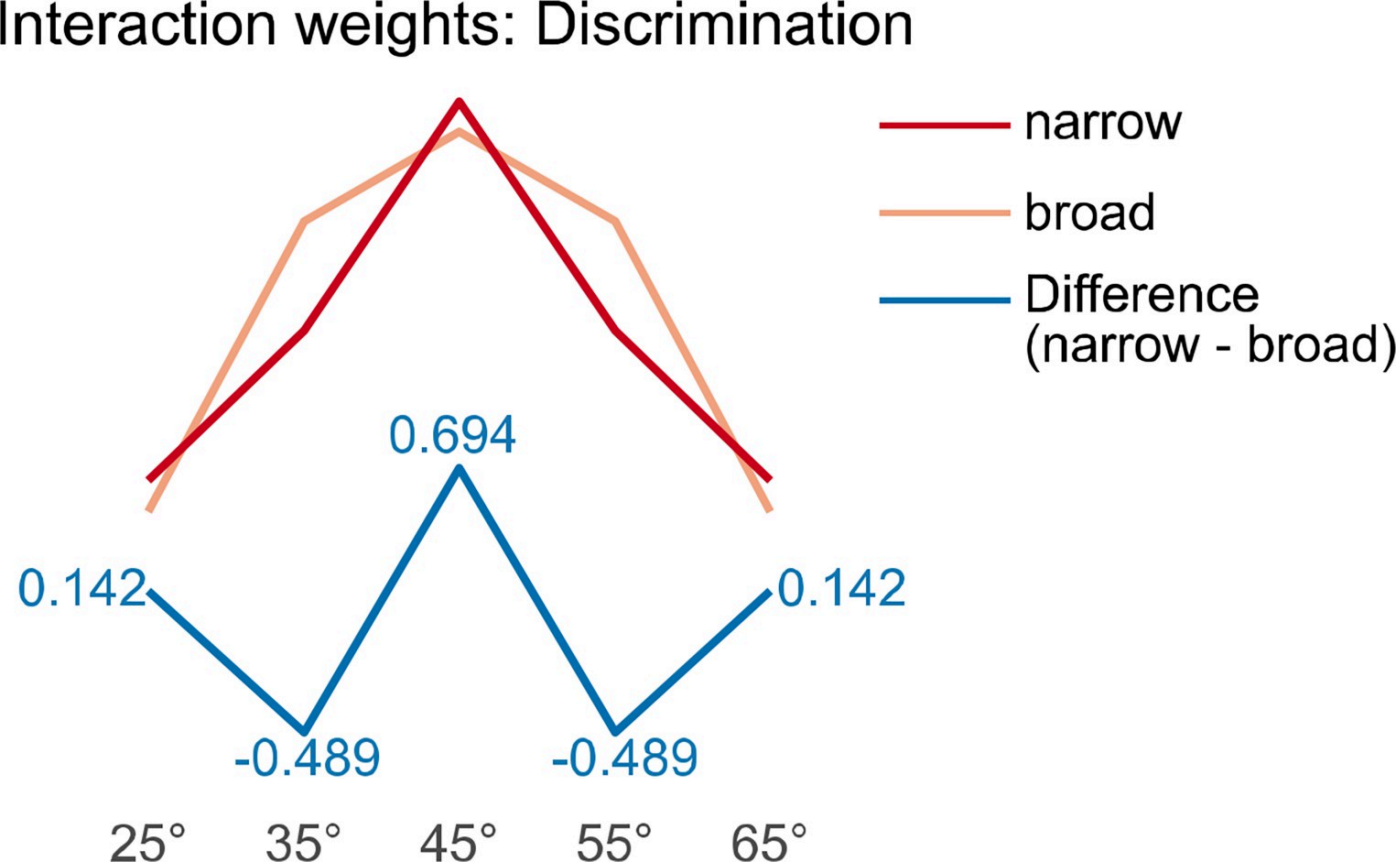
Contrast weights of Mexican hat for the orientation x synchronization interaction. Contrast weights for the expected discrimination to test the synchronization × orientation interaction in the theta-band. The weights shown for a narrow (red) and broad (orange) generalization pattern are examples that if subtracted (narrow–broad) produce the exact discrimination weights we used for the group × orientation interaction contrast (numbers in black font, 0.142, −0.498, 0.694, −0.498, 0.142; [20]), resembling a *Mexican Hat* (blue line).

Since the expected effect of phase-synchronized stimulation in the theta but not delta group could manifest in an altered generalization curve without changing the rating pattern dramatically, ANOVA interactions might not be able to detect pattern differences across CS gratings. Based on our previous study [20], for the theta-frequency band we expected a narrow generalization (i.e., higher ratings to the CS+ compared with the neighboring CS-) within the in-phase group and a broad generalization (i.e., high ratings to the CS+ and the neighboring CS-) in the out-of-phase group. The interaction between synchronization and orientation should therefore resemble a “W” or “Mexican hat pattern” (by subtracting a broad generalization from a narrow generalization; weights: 0.142, -0.489, 0.694, -0.489, and 0.142; [20]) when phase synchronization causes better discrimination between the CS+ and similar CS- gratings **(Fig 2)**. Since we hypothesized the discrimination ability depends on the frequency, we aimed at directly comparing the theta and delta group. In general, we expected a better discrimination in the theta compared with the delta group (orientation x frequency x synchronization interaction for contrast fits). Since we expected that the differences between in-phase and out-of-phase synchronization in the delta-frequency band to be smaller compared with the theta group, we pre-registered a planned test for another “Mexican hat” contrast fit of the orientation x synchronization x frequency interaction. The latter contrast results from subtracting a hypothetical “flat” contrast (no difference between in-phase and out-of-phase) in the delta group from the “Mexican hat” of the theta group. However, it was not possible to implement custom contrast weights for the comparison of two individual generalization patterns within the theta and delta group in SPSS. Therefore, in contrast to our pre-registered analysis, we decided to use the subsequently described discrimination indices (i.e., CS+ minus averaged CS-), as a similar measure of the discrimination ability, to calculate a 2 x 2 ANOVA, including the factors frequency (theta vs. delta) and synchronization (in-phase vs. out-of-phase). Differences between in-phase and out-of-phase in theta but not delta should manifest in a significant frequency x synchronization interaction. For a better comparability with our laboratory study, however, we nevertheless calculated the “Mexican hat” contrast fits for each frequency band separately.

Discrimination indices were calculated by subtracting the mean of all CS- gratings (i.e., 25°, 35°, 55°, 65°) from the reinforced CS+ (45°) orientation. Discrimination indices indicate a simple measure for the preference of CS+ against CS- gratings. For better comparability to our previous lab study [20], and as pre-registered, we z-transformed the discrimination indices, using the mean and standard deviation (*SD*) of discrimination indices across learning phases per participant.

Due to the fact that fear conditioning is subject to well-known sex differences, we conducted post hoc analyses including the factor *sex*. Hence, the 5 x 2 x 2 ANOVA was extended by a third between-subject factor *sex* (men vs. women), resulting in a 5 x 2 x 2 x 2 ANOVA. In case of significant differences including the additional factor, we subsequently conducted the 5 x 2 x 2 ANOVA within each sex. Further, “Mexican hat” contrast fits and discrimination indices were calculated separately for men and women, within the theta and delta frequency groups.

## Results

We first describe the fulfilment of the prerequisite (compliance control task) and report the success of fear acquisition and extinction. We then address the main (pre-registered) questions, i.e., rating differences depending on synchronization (in-phase vs. out-of-phase) and frequency band (theta vs. delta). Finally, explorative analyses are reported, including the factor sex. For an overview of all statistics, each statistical value reported in the following is additionally listed in **S2 Table**.

### Prerequisites: Compliance control and validation of web-based fear conditioning

Statistical analyses were conducted with data of those participants that passed the compliance control criteria, i.e., at least 50% correct identification of the number of beeps and the side of beep presentation. To check if this criterion helps separating participants that show learning from those that do not, we used the discrimination indices of the participants’ ratings (CS+ minus averaged CS- ratings) as dependent measures. We compared participants included (*N* = 162 [including 2 non-binary]; final sample comprised 80 men and 80 women, only) to participants excluded due to the compliance control task (*N* = 55). Arousal and valence ratings reflect the affective evaluation of the CS-US association. Thus, we used both measures collected after acquisition in a 2 x 2 ANOVA, with *rating measure* (valence vs. arousal) as a within-subject factor and *compliance* (passed compliance control: yes vs. no) as the between-subject factor (valence data were multiplied by -1 to reverse polarity). Analysis showed that participants who passed the compliance task had higher discrimination indices (valence, passed: *M* = 0.58 [*SD* = 0.80]; valence, failed: *M* = 0.06 [*SD* = 0.87]; arousal, passed: *M* = 0.60 [*SD* = 0.79]; arousal, failed: *M* = 0.16 [*SD =* 0.73], compliance main effect *F*(1,215) = 16.89, *p* < .001, $\eta_{p}^{2}$ = .145).

Similarly, the z-standardized CS-US contingency knowledge (= US-expectancy), showed a trend-level main effect of compliance (*F*(1,215) = 2.78, *p* = .097, $\eta_{p}^{2}$ = .013). However, when analyzing the discrimination index of raw US-expectancy ratings, the effect is even clearer (*F*(2,215) = 9.27, *p* = .003, $\eta_{p}^{2}$ = .041). Comparable with the affective ratings, discrimination indices were higher for participants that were included in our final sample (passed: *M* = 0.82 [*SD* = 0.95]; failed: *M* = 0.56 [*SD* = 1.04] for z-values).

**Fig 3:**
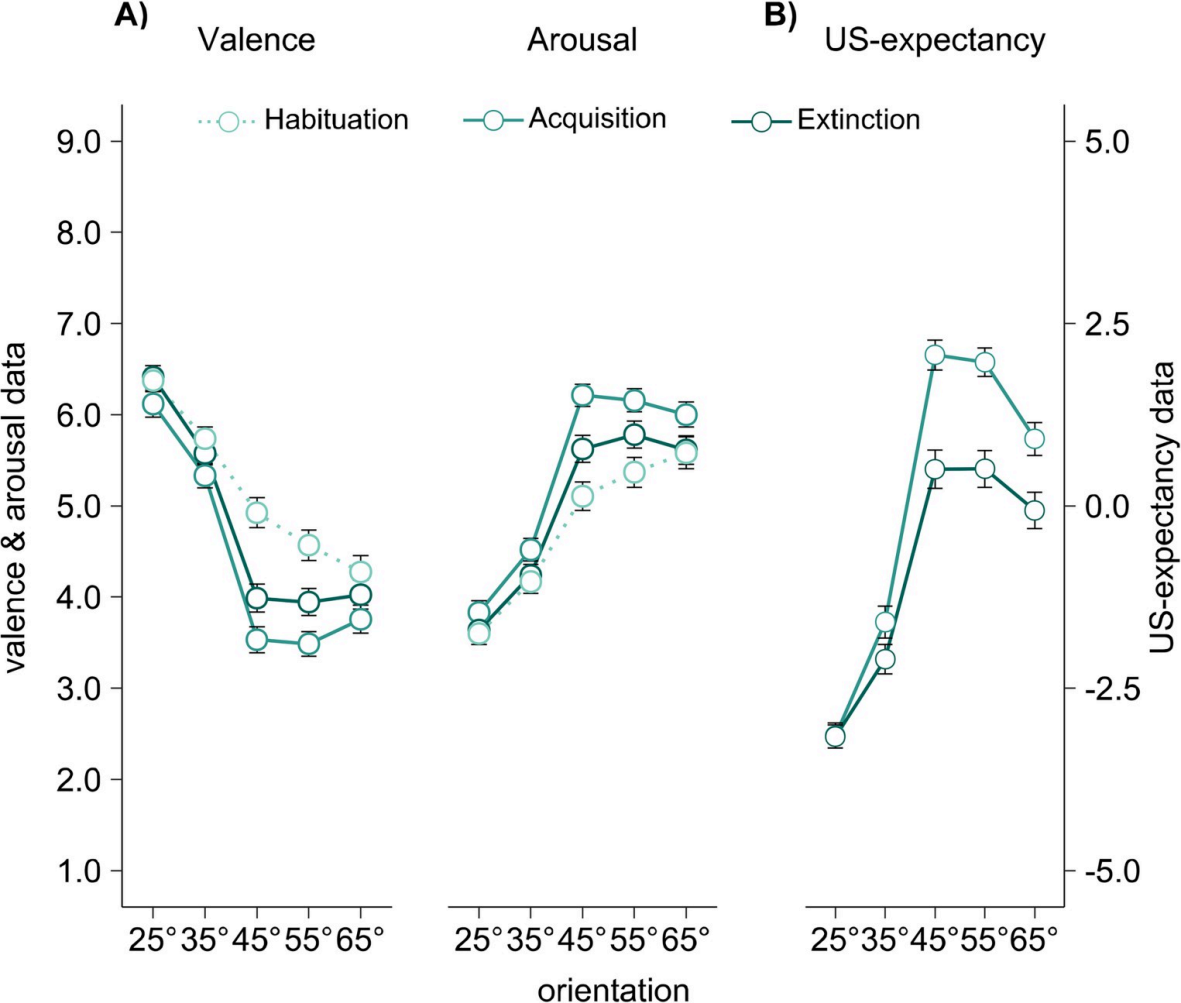
Validation of fear acquisition and extinction. Validation of fear acquisition and extinction for valence and arousal ratings **(A)** and US-expectancy ratings **(B)**. Valence of each CS grating was rated via Self-Assessment Manikins (SAM) on a 9-point scale from 1 (unpleasant) to 9 (pleasant). Similarly, arousal ratings were conducted via SAMs, here ranging from 1 (calm) to 9 (arousing) (see also *Materials and Methods section [Dependent variables])*. US expectancies after acquisition and extinction were rated on a scale from -5 (very certain, no US after this CS) over 0 (uncertain) to 5 (very certain, a US will follow this CS). Each data point represents averaged valence, arousal, or US-expectancy ratings, separately for acquisition and extinction but not differentiated for synchronization and frequency. Error bars show ±1 SEM.

Finally, on average, the discrimination indices were positive in the final sample (i.e., larger for the CS+ compared to the average of all CS-) for valence, arousal, and for US-expectancy ratings). This suggests successful acquisition in the web-based fear conditioning task. Further supporting successful acquisition, we found main effects of CS orientation in analysis including all CS orientations for valence (*F*(2.7, 414.0) = 111.19, *p* < .001, $\eta_{p}^{2}$ = .416 **Fig 3A** left panel), arousal (*F*(2.8, 431.1) = 107.17, *p* < .001, $\eta_{p}^{2}$ = .407, **Fig 3A** right panel) and US-expectancy (*F*(2.9, 452.3) = 140.24, *p* < .001, $\eta_{p}^{2}$ = .473, **Fig 3B**). A specific preference for the CS+ orientation, was confirmed by fitting generalization contrasts within all of the three measures (valence: *F*(1,156) = 88.80 *p* < .001, $\eta_{p}^{2}$ = .363; arousal: *F*(1,156) = 82.13, *p* < .001, $\eta_{p}^{2}$ = .345; US-expectancy: *F*(1,156) = 147.78, *p* < .001; $\eta_{p}^{2}$ = .486).

Extinction learning should manifest in decreasing rating intensity after extinction when comparing ratings after acquisition and after extinction. As expected, we found that the general levels of arousal, valence, and the expectation that an US occurs with one of the CS orientations, was significantly reduced after extinction (main effects of *LP* in valence(*F*(1,156) = 18.65, *p* < .001, $\eta_{p}^{2}$ = .107, arousal (*F*(1,156) = 19.80, *p* < .001, $\eta_{p}^{2}$ = .113, **Fig 3A** right panel, and US-expectancy *(F*(1,156) = 35.50, *p* < .001, $\eta_{p}^{2}$ = .185, **Fig 3B**).

In sum, our data strongly support that our online setting is suitable to successfully induce fear acquisition and extinction in a complex differential fear conditioning protocol with an auditory US.

### OSF-registered hypothesis

#### US-expectancy ratings

**Fig 4:**
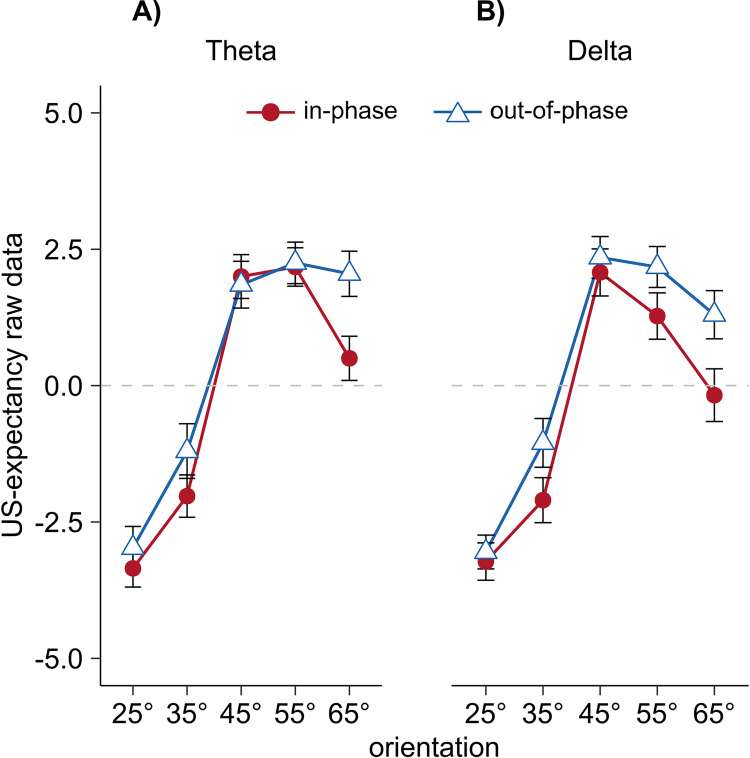
Raw US-expectancy data. Raw US-expectancy ratings separated for frequency (theta vs. delta) and synchronization condition (in-phase vs. out-of-phase). US-expectancies were collected as described in caption of **Fig 3** and the *Materials and Methods section (Dependent variables)*. Each data point represents mean US-expectancy ratings for each CS orientation over participants, separately for frequency and synchronization condition. Error bars show ±1 SEM. **S1 and S2 Figs** shows discrimination indices (CS+ minus the average of all CS-) and estimation statistics for z-transformed US-expectancy ratings. Discrimination indices were calculated for the comparison of in-phase vs. out-of-phase across frequency as well as separately for theta and delta frequency. The estimation plots in **S1 and S2 Figs** depict the estimation statistics including the individual values as well as effect sizes (Hedge’s g) as a bootstrap confidence interval (5000 samples [33]). **S1 Fig** shows the comparison of both synchronization conditions across the mean of theta and delta frequency, **S2 Fig** presents the comparison between in-phase and out-of-phase synchronization within each frequency (theta vs. delta).

Similar to our previous laboratory study [20], the current data revealed that synchronized input (in-phase groups) causes a narrower generalization of the US-expectancy ratings compared with asynchronized input (out-of-phase groups, main effect synchronization *F*(1,156) = 10.17, *p* = .002, $\eta_{p}^{2}$ = .061, **Fig 4**). However, it did not interact with the stimulation frequency (no synchronization x frequency interaction: *F*(1,156) = 0.34, *p* = .560, $\eta_{p}^{2}$ = .002, and no orientation x synchronization x frequency interaction: *F*(2.9,452.3) = 0.27, *p* = .838, $\eta_{p}^{2}$ = .002). Thus, contrary to our predictions, we did not find the expected theta-specific effect of synchronous CS+US presentation, also evident when comparing the discrimination indices (CS+ minus the mean of the CS- gratings) in dependence of synchronization and frequency (synchronization x frequency interaction for discrimination indices: *F*(1,156) = 0.42, *p* = .518, $\eta_{p}^{2}$ = .003). Accordingly, we did not find the Mexican hat contrast fit for the synchronization x orientation interaction when separately analyzing within the theta frequency group (*F*(1,78) = 0.40, *p* = .528, $\eta_{p}^{2}$ = .005) and delta frequency group (*F*(1,78) = 1.35, *p* = .249, $\eta_{p}^{2}$ = .005), respectively.

### Valence and arousal ratings

**Fig 5:**
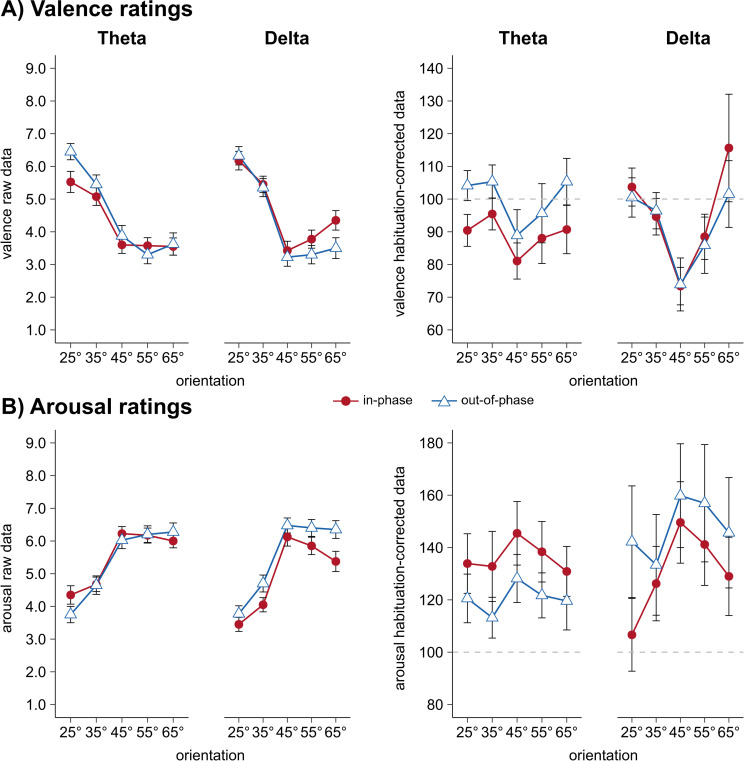
Raw and habituation-corrected valence and arousal ratings. Raw (left panel) as well as habituation-corrected (right panel) valence **(A)** and arousal **(B)** ratings separately for frequency (theta vs. delta) and synchronization condition (in-phase vs. out-of-phase). Valence ratings were collected as described in captions of **Fig 3** and the *Materials and Methods section (Dependent variables)*. Each data point represents mean valence ratings for each CS orientation over participants per frequency and synchronization condition. Error bars show ±1 SEM. Note that habituation-corrected values are depicted for better visualization of the fear generalization pattern. However, the statistics in the result section are based on the raw data. **S3 and S4 Figs** show discrimination indices (CS+ minus the average of all CS-) and estimation statistics for z-transformed valence and arousal ratings, respectively. For each frequency band (theta vs. delta) the discrimination index of in-phase and out-of-phase was compared.

Valence ratings confirm a successful learning of CS-US pairings, with the most negative valence ratings towards the 45° (CS+) orientation (see *Prerequisites*). However, we did not find the expected difference between in-phase and out-of-phase presentation, when accounting for the theta and delta frequency (orientation x synchronization x frequency interaction: *F*(2.6,414.0) = 0.39, *p* = .738, $\eta_{p}^{2}$ = .002, **Fig 5A**). Accordingly, discrimination indices did not differ between synchronization and frequency conditions (synchronization x frequency interaction: *F*(1,156) = 0.07, *p* = .798, $\eta_{p}^{2}$ = .000) and there was no fit of Mexican hat contrast weights analyzed for theta (*F*(1,78) = 1.36, *p* = .247, $\eta_{p}^{2}$ = .017) or delta frequency (*F*(1,78) = 0.05, *p* = .827, $\eta_{p}^{2}$ = .001, **Fig 5A**), separately.

As already shown, arousal data after acquisition show successful conditioning, with highest arousal ratings towards the CS+ (see results of *validation*). However, we did not find the expected orientation x synchronization x frequency interaction (*F*(2.8,431.1) = 0.14, *p* = .924, $\eta_{p}^{2}$ = .001, **Fig 5B**) and additionally no significant synchronization x frequency interaction in the discrimination indices (*F*(1,156) = 0.14, *p* = .710, $\eta_{p}^{2}$ = .001. This also became evident in a non-significant contrast fit of Mexican hat when comparing synchronization effects in theta (*F*(1,78) = 0.77, *p* = .384, $\eta_{p}^{2}$ = .010) or delta frequency (*F*(1,78) = 0.55, *p* = .463, $\eta_{p}^{2}$ = .007; **Fig 5B**).

In accordance with the ANOVA, estimation statistics of the comparison between in-phase and out-of-phase groups (calculated by subtracting the mean CS- from the CS+ orientation) did not reveal significant differences for valence **(S3 Fig)** and arousal ratings (**S4 Fig**).

### Explorative analysis

**Fig 6:**
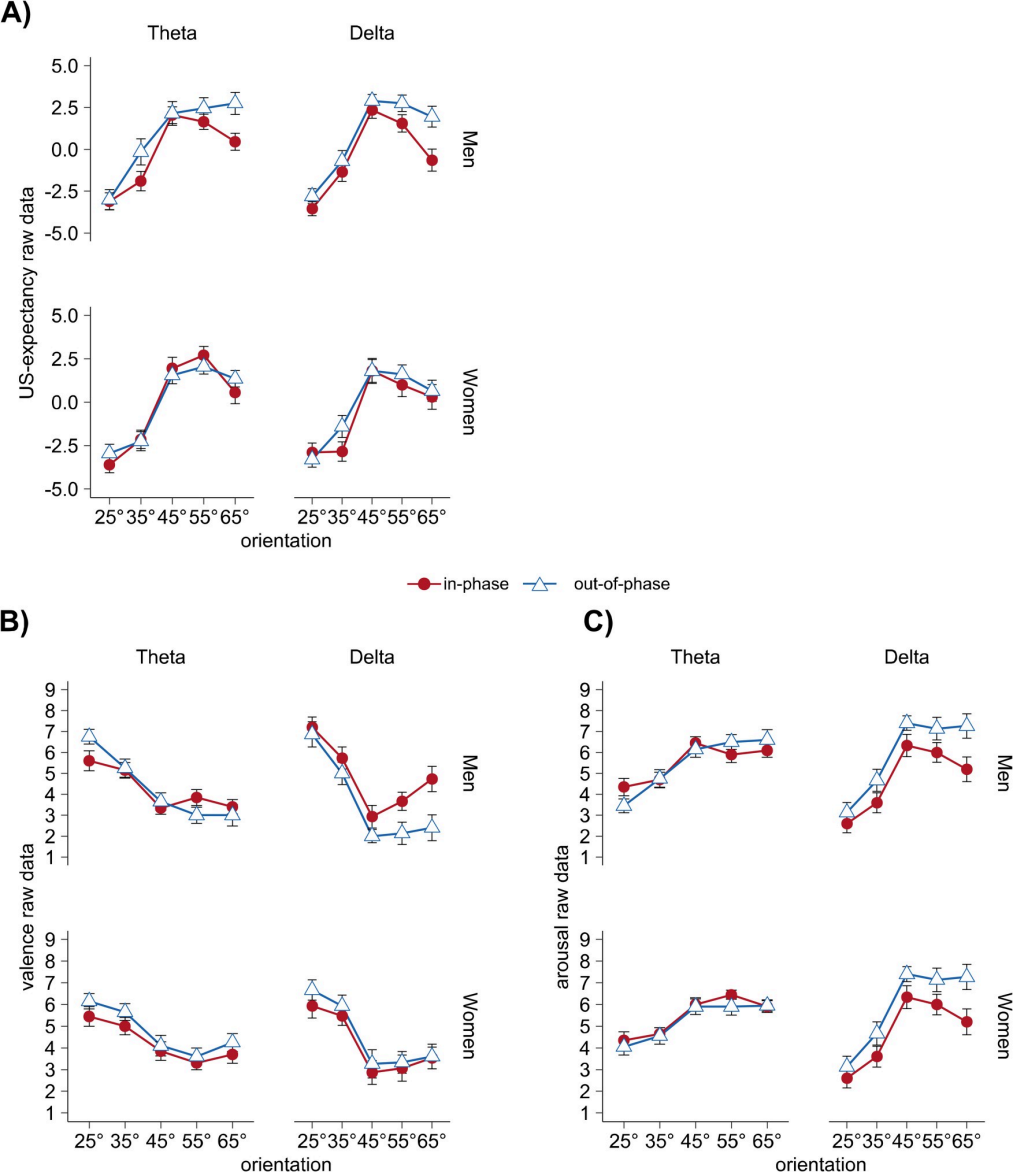
Valence, arousal, and US-expectancy data separately for men and women. US-expectancy ratings **(A)**, valence ratings **(B)**, and arousal ratings **(C)** after acquisition for each frequency (theta vs. delta) and synchronization (in-phase vs. out-of-phase), separated by sex: within each subplot, top row shows men (n = 80), bottom row shows women (n = 80). Each data point represents mean values for each CS orientation, separately for frequency, synchronization, and sex. Error bars show ±1 SEM. In order to improve the visualization of the fear generalization pattern, we corrected for the linear trend during habituation, acquisition, and extinction: **S5 Fig** shows these habituation-corrected valence and arousal rating.

Our planned sample size comprised the same number of male and female participants. For explorative purpose only, we therefore repeated our analysis by adding the factor sex (men vs. women). For US-expectancies after acquisition, we found a significant main effect of sex (*F*(1,152) = 6.06, *p* = .015, $\eta_{p}^{2}$ = .038) as well as a synchronization x sex interaction (*F*(1,152) = 4.47, *p* = .036, $\eta_{p}^{2}$ = .029) that was based on greater differences between in-phase and out-phase presentation in men (**Fig 6A**). In addition, we found a significant interaction of synchronization x sex in valence ratings (*F*(1,152) = 4.16, *p* = .043, $\eta_{p}^{2}$ = .027, **Fig 6B**) but not arousal ratings (synchronization x sex interaction: *F*(1,152) = 1.44, *p* = .232, $\eta_{p}^{2}$ = .009, **Fig 6C**).

Repeating the 5 x 2 x 2 ANOVA for men and women separately, supported the above pattern for valence rating with a trend-level interaction of frequency x synchronization (*F*(1,76) = 3.03, *p* = .086, $\eta_{p}^{2}$ = .038 in men (**Fig 6B** top panel), but not women (frequency x synchronization interaction: *F(*1,76*) =* 0.12, *p* = .726, $\eta_{p}^{2}$ = .002) **Fig 6B** bottom panel). With **Fig 6C** showing a similar difference between men and women for the arousal data, we found a comparable frequency x synchronization (trend-level) interaction in men (*F(*1,76) = 2.94 *p* = .090, $\eta_{p}^{2}$ = .037) as well as an additional trend-level main effect of synchronization (*F(*1,76) = 2.81 *p* = .098, $\eta_{p}^{2}$ = .036) in men only. Interestingly, men in the theta group showed the expected Mexican hat contrast fit with narrow generalization after in-phase presentation and broader generalization in the out-of-phase group (trend-level Mexican hat fit for orientation x synchronization interaction: *F*(1,38) = 3.49, *p* = .070, $\eta_{p}^{2}$ = .084). For the US-expectancy ratings, the separate analysis for men and women revealed a trend-level interaction of orientation x synchronization (*F*(2.9,222.5) = 2.61, *p* = .054, $\eta_{p}^{2}$ = .033) as well as a significant main effect of synchronization (*F*(1,76) = 11.57, *p* = .001, $\eta_{p}^{2}$ = .132) in men, but again, as seen in **Fig 6A**, women did not show comparable differences. For consistency across our measures, we also calculated the 2 x 2 ANOVA for the discrimination indices for men and women separately. However, due to the explorative character, they are only listed in the Supporting Information **S3 Table**.

In sum, in all three rating measures, the explorative analyses including the factor *sex* revealed more pronounced effects in men compared to women that depend on frequency and synchronization and partly (at least trendwise) interact with orientation and reveal the expected Mexican hat contrast fit in the theta-synchronization condition exclusively in men.

## Discussion

The current study aimed at 1) transferring previous findings of augmented affective ratings and CS-US contingency knowledge by theta-phase synchronized (vs. asynchronized) sensory input from a laboratory fear conditioning study to a web-based paradigm, and 2) expanding on our previous findings of this synchrony-induced augmentation by testing theta-specificity via a comparison with synchronization in the delta band. Based on our laboratory findings and the work of Clouter et al. [7], we hypothesized a theta-specific effect of phase synchronization that becomes apparent in improved learning of the aversive CS-US association. It should manifest in a better discrimination between the CS+ and most similar CS- orientations when CS and US gratings were presented phase-synchronized (vs. asynchronized) in theta while presentation in 1.7-Hz delta should not lead to differences between phase-synchronous and asynchronous stimulation.

In line with recent work of Stegmann et al. [23] and Björkstrand et al. [22], our findings support the ability to induce associative fear learning as well as extinction in a web-based fear conditioning paradigm. We further have to acknowledge that we used a complex generalization protocol. We found that the CS+ US association was successfully formed in terms of the increased arousal and unpleasantness ratings towards the CS+, that decreased gradually with decreasing similarity of the CS- gratings. Additionally, the knowledge about the CS-US contingency manifested in a similar learned generalization, with the highest US expectancy ratings for the CS+ and a gradual decrease towards the neighboring CS- orientations. Thus, in all three measures the web-based conditioning results were highly comparable to those of the published lab-based version [20]. Importantly, this was achieved with only minimal changes compared to the previously published task.

In a web-based study, task engagement and compliance are a major concern especially in a long-lasting, passive and aversive fear conditioning task using loud noise as US. In the web-based setting, participants have many distractions that are outside of experimental control, and many opportunities to disengage from the task or even avoid the aversive noise altogether. In order to keep the core task unchanged in the web-version, but assessing compliance effectively without violating the subjects’ privacy, we only introduced a very simple control task in the breaks between learning phases. Interestingly, this was sufficient to control for task engagement and unchanged auditory volume, as the results of our prerequisite analysis have shown. Moreover, our minimal compliance control task was a predictor of learning success.

Our results of enabling a rather complex fear generalization paradigm in a web-based approach, also emphasize the usability of discriminatory stimuli and open great opportunities for future (fear) research: Compared with laboratory studies, web-based designs have the advantage of being time-efficient and cost-effective. The time-efficiency also makes it a great tool for piloting data with new task designs. Additionally, a web-based design can potentially reach participants all over the world allowing the assessment of inter-cultural aspects that are difficult to include in one lab, and reducing bias from *testing western*, *educated*, *industrialized*, *rich*, *and democratic* (WEIRD) samples in psychology and neuroscience [34]. From the view of emotion research, web-based studies could also be a useful alternative when it comes to clinically relevant samples. While the typical study sample consists of healthy university students [35], participants that are confronted with symptoms of anxiety or phobias might avoid being part in a study that is conducted in a potentially stressful laboratory environment. The possibility of participating from home might help to collect data of so far underrepresented groups. On the other hand, it needs high responsibility in case of any decompensation.

While our data confirm fear acquisition and extinction, the expected effects of theta-phase specificity were ambiguous. Affective ratings of valence and arousal did not show any differences in generalization across the CS gratings that depended on phase synchronization or frequency. In contrast and as hypothesized, US-expectancy ratings that indicate the knowledge about the CS-US contingency revealed higher overall ratings in the out-of-phase group, suggesting that this group broadly generalizes across the CS orientations. Contrary to our expectations, however, this effect was independent of the frequency and hence, not specific for the theta-band. One possible reason for the discrepancy between the affective (valence and arousal) and cognitive (US expectancy) ratings concerns the perceived intensity of the aversive US. In both, our recent laboratory as well as the current web-based study, we instructed the participants to not only rate the affective quality of CS gratings but also asked for an evaluation of the valence and arousal of the auditory US after acquisition. Although both studies confirmed the US aversiveness by high arousal and low valence (i.e., unpleasant) ratings (laboratory study: arousal, *M* = 8.13, *SD* = 0.822; valence, *M* = 1.9, *SD* = 1.1; web-based study: arousal, *M* = 6.26, *SD* = 1.6; valence, *M* = 2.58, *SD* = 1.6), the comparison of both settings revealed lower arousal and unpleasantness (valence) ratings in the web-based study. This is probably based on the individual US-intensity adjustment in the current study. While the more declarative knowledge of CS-US contingency should be unaffected by lower US intensities, it might have an effect on emotional evaluation of CS valence and arousal, and may thus be less receptive for subtle differences in fear generalization. Future studies could try and avoid such problems by devising an even better procedure for the titration of US intensity and applying more rigorous control tasks (e.g., individualized near-threshold audio stimuli) to prevent participants from making even small changes in audio volume during the task.

The hypothesized theta-band specificity was based on overwhelming evidence in animal and human studies, revealing an involvement of theta synchronization in the communication between distinct brain regions and the coordination of neural activity [2, 3, 5, 6, 36, 37]. In the processing of fear and extinction memories, for example, theta oscillations synchronize within and between the main structures of the fear circuitry (i.e., amygdala, hippocampus, and parts of the medial prefrontal cortex [mPFC]), enabling precisely timed neural activity that is crucial for synaptic plasticity [16, for reviews see 38–42]. However, a new line of evidence suggests that low frequency (theta and delta) entrainment in general rather than theta-band specific entrainment might induce memory enhancing effects [43, 44]. Earlier studies already showed that slow delta frequency entrainment provides optimized windows for information processing in perceptual discrimination tasks in macaque monkeys [45] and improves reaction times in those primates and also humans [45, 46]. Interestingly, more recent findings revealed that the effects of slow-frequency entrainment might not be restricted to perception, but also play a role in higher-order cognition like memory formation [47]. Slow-frequency entrainment might provide an optimized neural rhythm to, for instance, coordinate higher frequencies (so-called cross-frequency phase-amplitude coupling), a mechanism that was repeatedly associated with memory processing [for a review see 47–50]. In a memory encoding and recognition task, Jones et al. [51] found a better recognition for those items that were presented rhythmically (fixed ITI) vs. arrhythmically (variable ITI) in a slow 1.67 Hz delta frequency. In accordance, visual target stimuli that were presented “on-beat” (synchronous) with an auditory 1.25 Hz background rhythm compared with “off-beat” (asynchronous) improved memory in a subsequent recognition task, suggesting that delta entrainment is effective in cross-modal memory processing [52]. Using a comparable paradigm, Hickey et al. [43] linked the improvement of memory to neural entrainment (measured as phase coherence and increased power at 1.25 Hz), showing that a greater entrainment during the encoding phase predicted a better subsequent memory. Taken together, there is growing evidence that low-frequency entrainment (delta-theta range) orchestrates neural activity to a degree that is supportive for memory encoding.

Another mechanism for better discrimination after in-phase vs. out-of-phase audio-visual sensory input may rely on an increase in salience of the synchronous stimuli via attentional mechanisms, irrespective of the specific stimulation frequency and oscillatory brain mechanisms, i.e., via synchronization per se. The temporal co-occurrence of auditory and visual features per se (e.g., onset/offset in our synchronous groups) may be a strong signal, indicating that the sound and visual signal are the same event, which in turn may trigger multisensory integration. Indeed, when presented (at least mainly) synchronously, even task unrelated, uninformative, transient auditory stimuli can amplify spatial [53–55] and feature-specific visual attention [56] and amplify visual processing. Such attentional gain may also explain our current finding of a narrower generalization of US-expectancy ratings in both the theta and delta synchronous groups. Stimuli that were viewed with higher attention could be easier to discriminate even in a temporally delayed rating. Our task design does not allow us to disentangle these two alternatives (i.e. low-frequency entrainment supporting memory encoding vs. audiovisual synchrony amplifying attention). Previous work from Clouter et al. [7] with 4 Hz stimulation showed that participants were unable to correctly identify the audio-visual condition as synchronous or asynchronous. This may speak against the argument that audiovisual synchrony amplified attention. However, earlier studies on the discriminability of multimodal synchrony vs. asynchrony suggest that most participants are easily able to discriminate audio-visual synchrony (vs. asynchrony), as long as the stimuli are not modulated with a frequency of more than 4 Hz [57, 58]. Our study lacks a rating of stimulus synchrony-asynchrony. Future studies could include this to help disentangle the underlying mechanisms. Of note, attentional amplification does not need to be reflected in a clear subjective distinction of the stimulus streams as synchronous or asynchronous. Finally, although there is an ongoing debate [56], one of the proposed mechanisms of multisensory effects on attention, action, and memory is synchronization of neural oscillations (but also phase resetting, see [59]). Thus, amplified attention for audio-visual synchrony and improved memory encoding via synchronization of low-frequency oscillations may in part share the same fundamental neurocomputational mechanism. Future neurostimulation and neurophysiological studies will hopefully improve our understanding of such mechanisms.

Given that fear conditioning is subject to well-known sex differences [e.g., 60–63], and in line with the higher prevalence of anxiety- and stress-related disorders in women [57–60], we conducted exploratory post-hoc analyses including the factor *sex*. Interestingly, in US-expectancy ratings only men responded differently to phase-synchronized vs. phase-asynchronized stimulation, independent of frequency. In contrast, women’s US-expectancy ratings generalized across the CS orientations, without showing any effects of synchronization or frequency. Valence and arousal ratings showed descriptively (but not significantly) similar sex difference. Future studies might further examine this preliminary evidence for a sex difference by studying a wider range of women, including free-cycling women (we only examined women taking oral contraceptives, with suppressed levels of endogenously produced 17β estradiol and progesterone).

With the current study we were able to create a “pandemic-friendly” alternative of a laboratory fear conditioning paradigm. However, conducting a web-based conditioning contains some important limitations, discussed below.

First, web-based studies are mainly restricted to ratings or response times. Although our previous findings were restricted to valence, arousal, and US-expectancy, utilizing well-established fear measures like skin conductance responses are generally useful to operationalize a successful fear acquisition and extinction response. Regarding the specific question of entraining theta or delta frequency either in-phase or out-of-phase, EEG steady-state stimulus evoked signals would also have been a validation of the induction of CS and US at a given frequency. This is specifically interesting for frequencies as low as delta (1.7 Hz) since studies using steady-state visually evoked potentials (ssVEPs) typically work with frequencies of 4 Hz or higher so far–most studies even use frequencies between 8–10 Hz [64, 65]. Nevertheless, recent evidence of memory-enhancing effects after stimulus presentation in a delta rhythm support the entrainment at these very low frequencies [43, 51, 52]. As a future perspective, our findings should also be compared to a higher frequency band like alpha, to specify the idea of a special role for low-frequency phase synchronization for the encoding of memory [47]. Of importance, the length of a single rhythmic cycle decreases with increasing frequency (e.g., one full cycle at 2 Hz lasts 500 ms, one cycle at 10 Hz lasts only 100 ms). Thus, with increasing frequency, the time window of high excitability, e.g., defined as one quarter of the full cycle, decreases [2]. We therefore suggest testing higher frequencies in a standardized laboratory setting, since small variances in timing, caused by different browsers or internet connection result in greater phase-lags variabilities with increasing frequencies.

Second, since participants conducted our study at home, we did not have the same control of the environment that usually comes with studies in a laboratory setting only. With this, the most important limitation for this study concerns the optimized timing of CS-US input. As reported by Bridges et al. [66], the precision of timing varies in dependence of the operating systems and the browser. Although we tried to minimize the effect by uploading the complete stimulus material on the participants’ PC at the beginning of the experiment and by instructing the participants to use one of the browsers that revealed the least deficits in timing, we were unable to control intra- or interindividual variance in the exact synchronization between the CS+ and US. However, even though the timing might not have been as exact as we planned, Fell & Axmacher [2] emphasizes that a lag of precisely 0 ms is not necessary for the induction of successful LTP. Instead, 10–20 ms delay of post-synaptic firing after activation of the presynaptic neuron should be sufficient. Nevertheless, future studies should consider to systematically validate the stimulus timings by assessing intra- and interindividual variability of timings in the coded experiment for different operating systems, browsers, and internet connections to further strengthen the results.

Third, although the conducted compliance control task enabled us to proof the presence and general compliance towards the experiment, we cannot rule out that the participants distracted themselves during the aversive conditioning procedure. Importantly, this did obviously not influence the formation of the CS-US association per se.

Fourth, cross-trial temporal consistency of the phase shift between the CS+ and the US varied due to the 90°, 180°, 270° lags in the out-of-phase group, while it persisted at 0° in the in-phase groups. We cannot rule out that the subtle temporal variations within the out-of-phase condition (compared to the in-phase condition) might have led to perceptual differences between both groups that resulted in broader vs. narrower generalization patterns across the CS+. Nevertheless, previous studies examining episodic memory did not find learning differences between the 90°, 180°, 270° variation [7] or restricted the out-of-phase presentation to a 180° shift only [8]. In accordance with [8], future fear conditioning studies that specifically focus on generalization across perceptually similar CSs should better use a single phase shift in the out of phase group (i.e., 180° only) to avoid variability of CS-US shifting within the asynchronous condition.

Finally, an interesting—although from our perspective unfortunate—aspect is the strong linear relation across CS orientations prior to any experimental manipulation (i.e., in the habituation phase) as well as after acquisition and extinction. While we found evidence for affective evaluations that differed in dependence of the orientation of contour features in the literature [67], it is contrary to our findings: long horizontal contours were reported to be related to judgements of openness and depth, hence associated with safety and pleasantness. In contrast, vertical lines were related to an environment including long grass and trees that might hide potential danger [67]. Although we did not use perfectly horizontal or vertical orientations, the extremely robust orientation effect we found during habituation in all our ratings might be an interesting starting point for further studies.

In sum, the current study provides an example of how to use a complex generalization fear conditioning design in a web-based study. While we found robust fear acquisition and extinction, the ambiguous findings of synchronization or frequency effects suggest that low frequency rather than theta-specific entrainment supports the (predominantly declarative) memory of CS-US contingency. However, the limitations that come with the web-based approach underlines that time-critical questions might have greater success in a controllable laboratory environment. Nevertheless, from a methodological perspective, our study emphasizes some aspects that should be considered: when it comes to the US, using individually adjustable US-intensity is a great way to ensure a sufficient US aversiveness. In addition, we recommend the use of a compliance control task to check the presence and active participation. Besides, the choice of a low-volume beep sounds gave us a further guarantee that participants followed the instructions during the US-intensity adjustment. Hence, without prior notice, we added a control task that simultaneously tested for visual and auditory compliance. Importantly, despite these discussed limitations, the current study emphasizes augmented discriminations in the declarative knowledge of CS-US contingency when frequency-modulated stimuli are presented phase-synchronized (compared with phase-asynchronized) at a low frequency, i.e., our findings were not specific for the theta-frequency band. Interestingly, however, exploratory analyses showed theta-specific augmented discrimination became evident in US-expectancies in men, not women. Hence, future studies should include male and female participants.

## Supporting information

S1 Dataset Anonymized dataset, including raw and z-transformed ratings of US-expectancy, valence, and arousal. Dataset includes raw valence (val), arousal (aro), and US-expectancy (exp) ratings, as well as z-standardized ratings and discrimination indices (disidx). *Subject ID* is the anonymized subject identification number, *date* shows the date of participation, *val*:*US* and *aro_US* represents the valence and arousal rating of the auditory US that was assessed after acquisition. The learning phases are abbreviated by *hab* for habituation, *acq* for acquisition, and *ext* for extinction. Each grating orientation is shown by the value between *25* and *65*.
(XLSX)

S1 Table Means and common SDs used to calculate power analysis for US-expectancy, valence, and arousal ratings. (DOCX)

S2 Table Summary of statistical analyses. Table shows statistical analyses including *p* value and effect size for each rating (US-expectancy, valence, arousal).
(DOCX)

S3 Table Discrimination indices for explorative analysis, including the factor sex. For ratings after acquisition, discrimination indices (CS+ minus averaged CS-) are calculated to assess differences in the ability to discriminate the CS+ and adjacent CS- gratings between synchronization conditions (in-phase vs. out-of-phase) and frequency (theta vs. delta). Indices were used in a 2 x 2 ANOVA, including the between-subject factors *synchronization* and *frequency* for men and women separately. Within each ratings measure, the table lists the main effect of frequency, the main effect of synchronization, and the interaction between synchronization and frequency. For valence and arousal, as well as US-expectancies, discrimination indices are presented as z-values.
(DOCX)

S1 Fig Discrimination indices for US-expectancy ratings, independent of frequency. The discrimination index was computed as the difference between the reinforced 45° orientation (CS+) grating and the average of the four CS–orientations. Data and effect sizes are shown as a Cumming estimation plot (http://www.estimationstats.com). Left column, Swarm plots show the z-standardized discrimination indices independent of frequency (each dot is the discrimination index of one participant). Group statistics are indicated to the right of each swarm as gapped lines (gap = mean, line length  =  1 SD). Right column, Effect size estimates (Hedges’ *g*, black dots) for the comparison between in-phase vs out-of-phase, across theta and delta frequency and their 95% confidence interval (CI; vertical error bars). The unpaired Hedge’s *g* of out-of-phase (n = 80) minus in-phase (n = 80): -0.29 [95% CI, -0.594, 0.0298]. The 5000 bootstrap samples were taken for CI estimation; the CI is bias corrected and accelerated.
(TIF)

S2 Fig Discrimination indices for US-expectancy ratings within the theta and delta frequency. The discrimination index was computed as the difference between the reinforced 45° orientation (CS+) grating and the average of the four CS–orientations. Data and effect sizes are shown as a Cumming estimation plot (http://www.estimationstats.com). Top row, Swarm plots show the z-standardized discrimination indices per frequency (each dot is the discrimination index of one participant). Group statistics are indicated to the right of each swarm as gapped lines (gap = mean, line length  =  1 SD). Bottom row, Effect size estimates (Hedges’ *g*, black dots) for the relevant comparisons (in-phase vs out-of-phase within theta and delta frequency) and their 95% confidence interval (CI; vertical error bars). The unpaired Hedge’s *g*: for the theta frequency our-of-phase (n = 40) minus Theta in-phase (n = 40): –0.403 [95% CI, -0.855, 0.0389]; for the delta frequency out-of-phase (n = 40) minus in-phase (n = 40): -0.18 [95% CI, –0.619, 0.273]. The 5000 bootstrap samples were taken for CI estimation; the CI is bias corrected and accelerated.
(TIF)

S3 Fig Discrimination indices for valence ratings. The discrimination index was computed as the difference between the reinforced 45° orientation (CS+) grating and the average of the four CS–orientations. Data and effect sizes are shown as a Cumming estimation plot (http://www.estimationstats.com). Top row, Swarm plots show the z-standardized discrimination indices per frequency (each dot is the discrimination index of one participant). Group statistics are indicated to the right of each swarm as gapped lines (gap = mean, line length  =  1 SD). Bottom row, Effect size estimates (Hedges’ *g*, black dots) for the relevant comparisons (in-phase vs out-of-phase within theta and delta frequency) and their 95% confidence interval (CI; vertical error bars). The unpaired Hedge’s *g*: for the theta frequency out-of-phase (n = 40) minus theta in-phase (n = 40): 0.11 [95% CI, -0.327, 0.538]; for the delta frequency out-of-phase (n = 40) minus in-phase (n = 40): 0.259 [95% CI, -0.207, 0.665]. The 5000 bootstrap samples were taken for CI estimation; the CI is bias corrected and accelerated.
(TIF)

S4 Fig Discrimination indices for arousal ratings. The discrimination index was computed as the difference between the reinforced 45° orientation (CS+) grating and the average of the four CS–orientations. Data and effect sizes are shown as a Cumming estimation plot (http://www.estimationstats.com). Top row, Swarm plots show the z-standardized discrimination indices per frequency (each dot is the discrimination index of one participant). Group statistics are indicated to the right of each swarm as gapped lines (gap = mean, line length  =  1 SD). Bottom row, Effect size estimates (Hedges’ *g*, black dots) for the relevant comparisons (in-phase vs out-of-phase within theta and delta frequency) and their 95% confidence interval (CI; vertical error bars). The unpaired Hedge’s *g*: for the theta frequency our-of-phase (n = 40) minus theta in-phase (n = 40): -0.204 [95% CI, -0.643, 0.24]; for the delta frequency out-of-phase (n = 40) minus in-phase (n = 40): -0.212 [95% CI, -0.639, 0.239]. The 5000 bootstrap samples were taken for CI estimation; the CI is bias corrected and accelerated.
(TIF)

S5 Fig Habituation-corrected valence and arousal data separately for men and women. To improve the visualization of the fear generalization pattern for men and women separately, we corrected for the linear trend during habituation, acquisition, and extinction, conducting a habituation correction for valence **(A)** and arousal **(B)** data. Both valence and arousal data show ratings after acquisition for each frequency (theta vs. delta) and synchronization (in-phase vs. out-of-phase), separated by sex: within each subplot, top row shows men (n = 80), bottom row shows women (n = 80). Each data point represents mean, habituation-corrected values for each CS orientation, separately for frequency, synchronization, and sex. Error bars show ±1 SEM.
(TIF)
