# Supplementary material for: Effects of phase synchronization and frequency specificity in the encoding of conditioned fear–a web-based fear conditioning study
Source: PLoS One. 2023 Mar 3;18(3):e0281644. doi: 10.1371/journal.pone.0281644 (PMC9983861; doi:10.1371/journal.pone.0281644)
Supplement: S1 Table — (DOCX) [file pone.0281644.s002.docx]

| S1 Table. Means and common standard deviations for power analysis based on [20]. | | | |
| --- | --- | --- | --- |
|  | **US-expectancy ratings** | **Arousal ratings** | **Valence ratings** |
| **Means for each cell in the design** | | | |
| In-sync, theta, 25° | -2.2 | 4.45 | 5.55 |
| In-sync, theta, 35° | -0.85 | 5.05 | 4.9 |
| In-sync, theta, 45° | 2.5 | 6.95 | 3.55 |
| In-sync, theta, 55° | -0.55 | 5.25 | 5.15 |
| In-sync, theta, 65° | -2 | 4.15 | 5.7 |
| In-sync, delta, 25° | -0.45 | 5.35 | 4.65 |
| In-sync, delta, 35° | 1.6 | 5.85 | 4.05 |
| In-sync, delta, 45° | 2.4 | 6.3 | 4.05 |
| In-sync, delta, 55° | 0.7 | 5.85 | 4.45 |
| In-sync, delta, 65° | -1.4 | 4.8 | 5.4 |
| A-sync, theta, 25° | -0.45 | 5.35 | 4.65 |
| A-sync, theta, 35° | 1.6 | 5.85 | 4.05 |
| A-sync, theta, 45° | 2.4 | 6.3 | 4.05 |
| A-sync, theta, 55° | 0.7 | 5.85 | 4.45 |
| A-sync, theta, 65° | -1.4 | 4.8 | 5.4 |
| A-sync, delta, 25° | -0.45 | 5.35 | 5.65 |
| A-sync, delta, 35° | 1.6 | 5.85 | 4.05 |
| A-sync, delta, 45° | 2.4 | 6.3 | 4.05 |
| A-sync, delta, 55° | 0.7 | 5.85 | 4.45 |
| A-sync, delta, 65° | -1.4 | 6.3 | 5.4 |
| **Common SDs** | | | |
|  | 1.3 | 1.3 | 1.3 |
