# Supplementary material for: Effects of phase synchronization and frequency specificity in the encoding of conditioned fear–a web-based fear conditioning study
Source: PLoS One. 2023 Mar 3;18(3):e0281644. doi: 10.1371/journal.pone.0281644 (PMC9983861; doi:10.1371/journal.pone.0281644)
Supplement: S2 Table — Table shows statistical analyses including p value and effect size for each rating (US-expectancy, valence, arousal). (DOCX) [file pone.0281644.s003.docx]

|  | **S2 Table. Summary of statistical analyses.**  Table shows statistical analyses including *p* value and effect size for each rating (US-expectancy, valence, arousal). | | | | | | |
| --- | --- | --- | --- | --- | --- | --- | --- |
|  |  | **DV & Learning-phase** | **Test** | **Effects** | **Statistics** | ***p-*value** | **Effect size (ƞ^2^p)** |
|  | **Prerequisite: Compliance Control Task** | | | | |  |  |
|  |  | *Valence & Arousal* | |  |  |  |  |
|  |  |  | ANOVA | ME C | *F*_(1,215)_ = 16.89 | <.001 | .145 |
|  |  |  | ANOVA | ME AR | *F*_(1,215)_ = 1.97 | .162 | .009 |
|  |  |  | ANOVA | INT C x AR | *F*_(1,215)_ = 0.78 | .379 | .004 |
|  |  | *US expectancy* | |  |  |  |  |
|  |  |  | ANOVA_z-values_ | ME C | *F*_(1,215)_ = 2.78 | .097 | .013 |
|  |  |  | ANOVA_raws_ | ME C | *F*_(1,215)_ = 9.27 | .003 | .041 |
|  | **Validation: Acquisition and extinction** | | | | | | |
|  |  | *Acquisition* |  |  |  |  |  |
|  |  |  | ANOVA_Val_ | ME O | *F*_(2.7,414.0)_ = 111.19 | <.001 | .416 |
|  |  |  | ANOVA_Aro_ | ME O | *F*_(2.8,431.1)_ = 107.17 | <.001 | .407 |
|  |  |  | ANOVA_US-exp_ | ME O | *F*_(2.9,452.3)_ = 140.24 | <.001 | .473 |
|  |  |  | ANOVA_Val_ | Gen | *F*_(1,156)_ = 88.80 | <.001 | .363 |
|  |  |  | ANOVA_Aro_ | Gen | *F*_(1,156)_ = 82.13 | <.001 | .345 |
|  |  |  | ANOVA_US-exp_ | Gen | *F*_(1,156)_ = 147.78 | <.001 | .486 |
|  |  | *Acq vs. Ext* | ANOVA_Val_ | ME LP | *F*_(1,156)_ = 18.65 | <.001 | .107 |
|  |  |  | ANOVA_Aro_ | ME LP | *F*_(1,156)_ = 19.80 | <.001 | .113 |
|  |  |  | ANOVA_US-exp_ | ME LP | *F*_(1,156)_ = 35.50 | <.001 | .185 |
|  | **OSF-registered analyses** | | | |  |  |  |
|  |  | *US-expectancy (after acquisition)* | | |  |  |  |
|  |  |  | ANOVA | ME S* | *F*_(1,156)_ = 10.17 | .002 | .061 |
|  |  |  | ANOVA | INT F x S | *F*_(1,156)_ = 0.34 | .560 | .002 |
|  |  |  | ANOVA | ME O x F x S | *F*_(2.9,452.3)_ = 0.27 | .838 | .002 |
|  |  |  | ANOVA_Index_ | INT S x F | *F*_(1,156)_ = 0.42 | .518 | .003 |
|  |  |  | ANOVA | „Mex“ (Theta) INT O x S | *F*_(1,78)_ = 0.40 | .528 | .005 |
|  |  |  | ANOVA | „Mex“ (Delta)  INT O x S | *F*_(1,78)_ = 1.35 | .249 | .017 |
|  |  | *Valence (after acquisition)* | | | |  |  |
|  |  |  | ANOVA | ME O x F x S | *F*_(2.6,414.0)_ = 0.39 | .738 | .002 |
|  |  |  | ANOVA_Index_ | INT S x F | *F*_(1,156)_ = 0.07 | .798 | .000 |
|  |  |  | ANOVA | „Mex“ (Theta) INT O x S | *F*_(1,78)_ = 1.36 | .247 | .017 |
|  |  |  | ANOVA | „Mex“ (Delta) INT O x S | *F*_(1,78)_ = 0.05 | .827 | .001 |
|  |  | *Arousal (after acquisition)* | | | |  |  |
|  |  |  | ANOVA | ME O x F x S | *F*_(2.8,431.1)_ = 0.14 | .924 | .001 |
|  |  |  | ANOVA_Index_ | INT S x F | *F*_(1,156)_ = 0.14 | .710 | .001 |
|  |  |  | ANOVA | „Mex“ (Theta) INT O x S | *F*_(1,78)_ = 0.77 | .384 | .010 |
|  |  |  | ANOVA | „Mex“ (Delta)  INT O x S | *F*_(1,78)_ = 0.55 | .463 | .007 |
|  | **Explorative Analyses (including the factor *sex)*** | | | | | |  |
|  |  | *5 x 2 x 2 x 2 ANOVA* | |  |  |  |  |
|  |  |  | ANOVA_US-exp_ | ME Sex | *F*_(1,152)_ = 6.06 | .015 | .038 |
|  |  |  | ANOVA_US-exp_ | S x Sex | *F*_(1,152)_ = 4.47 | .036 | .029 |
|  |  |  | ANOVA_val_ | S x Sex | *F*_(1,152)_ = 4.16 | .043 | .027 |
|  |  |  | ANOVA_Aro_ | S x Sex | *F*_(1,152)_ = 1.44 | .232 | .009 |
|  |  | *5 x 2 x 2 ANOVA* | |  |  |  |  |
|  |  | *Men* | ANOVA_val_ | INT F x S | *F*_(1,76)_ = 3.03 | .086 | .038 |
|  |  | *Women* | ANOVA_val_ | INT F x S | *F*_(1,76)_ = 0.12 | .726 | .002 |
|  |  | *Men* | ANOVA_Aro_ | INT F x S | *F*_(1.76)_ = 2.94 | .090 | .037 |
|  |  |  | ANOVA_Aro_ | ME S | *F*_(1,76)_ = 2.81 | .098 | .036 |
|  |  |  | ANOVA_Aro_ | „Mex“ (Theta)  INT O x S | *F*_(1,38)_ = 3.49 | .070 | .084 |
|  |  | *Men* | ANOVA_US-exp_ | INT O x S | *F*_(2.9,222.5)_ = 2.61 | .054 | .033 |
|  |  |  | ANOVA_US-exp_ | ME S | *F*_(1,76)_ = 11.57 | .001 | .132 |
|  |  | *Women* | ANOVA_US-exp_ | INT O x S | *F*_(2.7,208.3)_ = 0.43 | .714 | .006 |
|  |  |  | ANOVA_US-exp_ | ME S | *F*_(1,76)_ = 0.87 | .011 | .011 |

Notes: AR = affective ratings; Aro = Arousal ratings; ANOVA = repeated-measures ANOVA; C = compliance; DV = dependent variable; F = frequency; Gen = generalization contrast fit; Index = Discrimination Index (CS+ minus averaged CS-); INT = interaction; LP = learning phase; ME = main effect; Mex = Mexican hat contrast fit; ƞ^2^p = partial ƞ^2^; O = orientation; raw = raw values; S = synchronization; US-exp = US-Expectancy ratings; Val = Valence ratings; z-values = z-standardized values

*within the OSF pre-registration, we did not expect the synchronization effect to be independent of the factor frequency.
