# Supplementary material for: Effects of phase synchronization and frequency specificity in the encoding of conditioned fear–a web-based fear conditioning study
Source: PLoS One. 2023 Mar 3;18(3):e0281644. doi: 10.1371/journal.pone.0281644 (PMC9983861; doi:10.1371/journal.pone.0281644)
Supplement: S3 Table — For ratings after acquisition, discrimination indices (CS+ minus averaged CS-) are calculated to assess differences in the ability to discriminate the CS+ and adjacent CS- gratings between synchronization conditions (in-phase vs. out-of-phase) and frequency (theta vs. delta). Indices were used in a 2 x 2 ANOVA, including the between-subject factors synchronization and frequency for men and women separately. Within each ratings measure, the table lists the main effect of frequency, the main effect of synchronization, and the interaction between synchronization and frequency. For valence and arousal, as well as US-expectancies, discrimination indices are presented as z-values. (DOCX) [file pone.0281644.s004.docx]

| **S3 Table.** **Discrimination indices (z-transformed) for explorative analysis, including the factor sex.** | | | |
| --- | --- | --- | --- |
|  |  | **Effects** | **Statistics** |
|  | **Valence ratings** | | |
|  |  | *Men* | |
|  |  | Main effect frequency | *F*_(1,76)_ = 4.34, *p* = .041, ƞ^2^p = 054 |
|  |  | Main effect synchronization | *F*_(1,76)_ = 1.64, *p* = .204, ƞ^2^p = .021 |
|  |  | Synchronization x frequency interaction | *F*_(1,76)_ = 0.07, *p* = .790, ƞ^2^p = .001 |
|  |  | *Women* | |
|  |  | Main effect frequency | *F*_(1,76)_ = 2.06, *p* = .155, ƞ^2^p = .026 |
|  |  | Main effect synchronization | *F*_(1,76)_ = 0.28, *p* = .597, ƞ^2^p = .004 |
|  |  | Synchronization x frequency interaction | *F*_(1,76)_ = .0.38, *p* = .541, ƞ^2^p = .005 |
|  | **Arousal ratings** | | |
|  |  | *Men* |  |
|  |  | Main effect frequency | *F*_(1,76)_ = 1.06, *p* = .306, ƞ^2^p = .014 |
|  |  | Main effect synchronization | *F*_(1,76)_ = 0.76, *p* = .388, ƞ^2^p = .010 |
|  |  | Synchronization x frequency interaction | *F*_(1,76)_ = 0.02, *p* = .896, ƞ^2^p = .000 |
|  |  | *Women* |  |
|  |  | Main effect frequency | *F*_(1,76)_ = 0.61, *p* = .437, ƞ^2^p = .008 |
|  |  | Main effect synchronization | *F*_(1,76)_ = 1.04, *p* = .311, ƞ^2^p = .014 |
|  |  | Synchronization x frequency interaction | *F*_(1,76)_ = 0.31, *p* = .578, ƞ^2^p = .004 |
|  | **US-expectancy ratings** | | |
|  |  | *Men* |  |
|  |  | Main effect frequency | *F*_(1,76)_ = 1.227, *p* = .272, ƞ^2^p = .016 |
|  |  | Main effect synchronization | *F*_(1,76)_ = 4:615, *p* = .035, ƞ^2^p = .057 |
|  |  | Synchronization x frequency interaction | *F*_(1,76)_ = 0:555, *p* = .459, ƞ^2^p = .007 |
|  |  | *Women* |  |
|  |  | Main effect frequency | *F*_(1,76)_ = 0:094, *p* = .760, ƞ^2^p = .001 |
|  |  | Main effect synchronization | *F*_(1,76)_ = 0:423, *p* = .542, ƞ^2^p = .005 |
|  |  | Synchronization x frequency interaction | *F*_(1,76)_ = 0:051, *p* = .821, ƞ^2^p = .001 |
