# Supplementary figures and images for: Effects of phase synchronization and frequency specificity in the encoding of conditioned fear–a web-based fear conditioning study
Source: PLoS One. 2023 Mar 3;18(3):e0281644. doi: 10.1371/journal.pone.0281644 (PMC9983861; doi:10.1371/journal.pone.0281644)

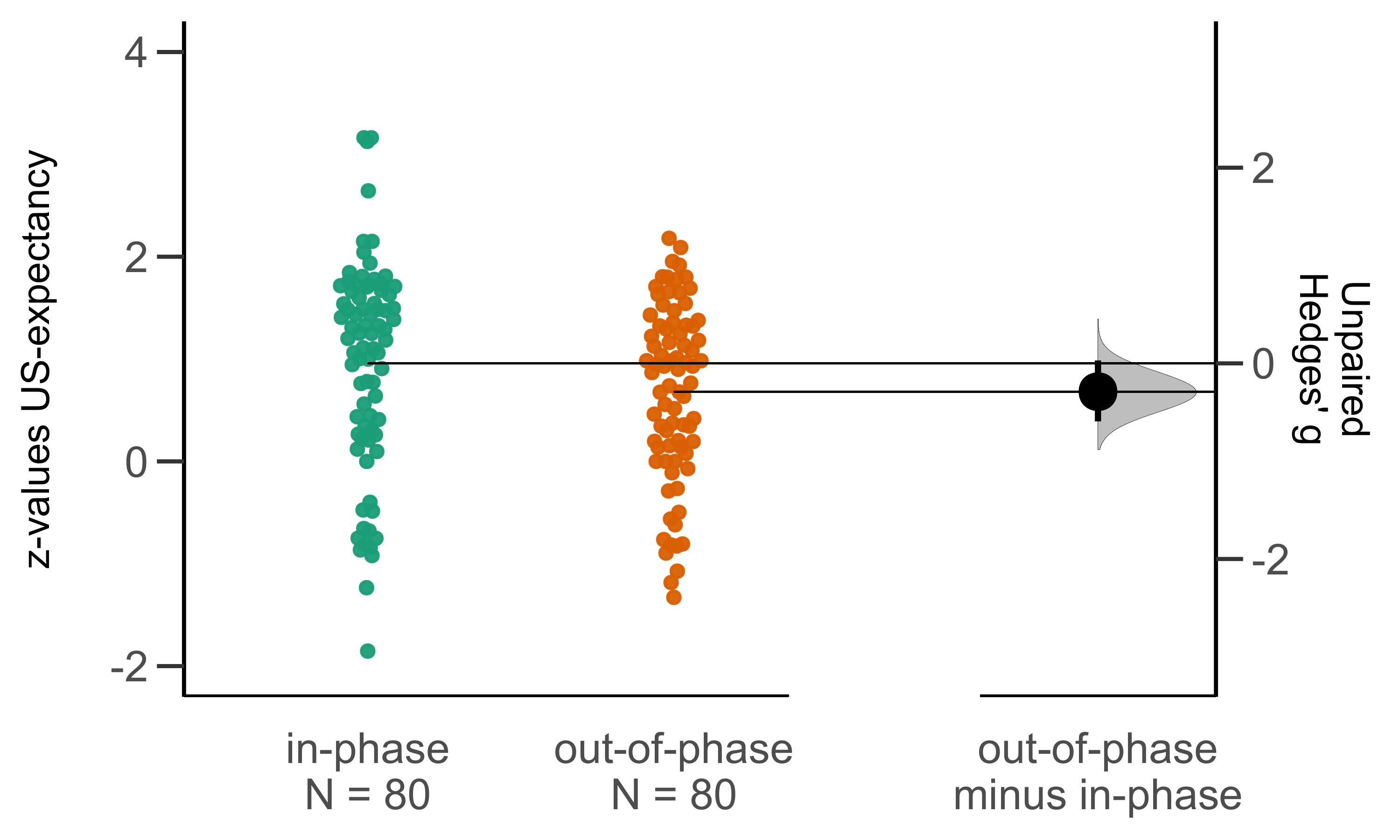

Supplement: S1 Fig — The discrimination index was computed as the difference between the reinforced 45° orientation (CS+) grating and the average of the four CS–orientations. Data and effect sizes are shown as a Cumming estimation plot (http://www.estimationstats.com). Left column, Swarm plots show the z-standardized discrimination indices independent of frequency (each dot is the discrimination index of one participant). Group statistics are indicated to the right of each swarm as gapped lines (gap = mean, line length = 1 SD). Right column, Effect size estimates (Hedges’ g, black dots) for the comparison between in-phase vs out-of-phase, across theta and delta frequency and their 95% confidence interval (CI; vertical error bars). The unpaired Hedge’s g of out-of-phase (n = 80) minus in-phase (n = 80): -0.29 [95% CI, -0.594, 0.0298]. The 5000 bootstrap samples were taken for CI estimation; the CI is bias corrected and accelerated. (TIF) [file pone.0281644.s005.tif]

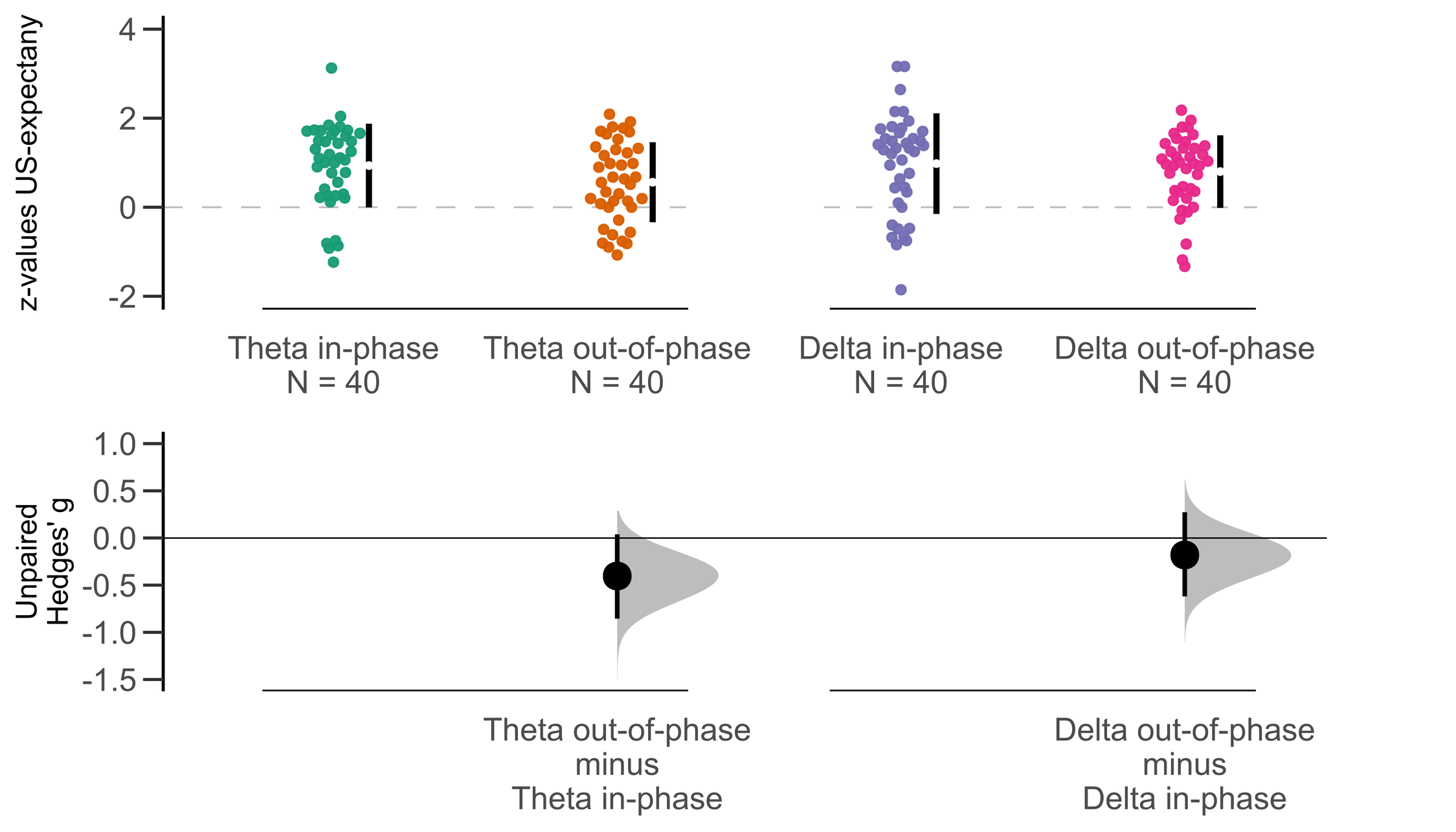

Supplement: S2 Fig — The discrimination index was computed as the difference between the reinforced 45° orientation (CS+) grating and the average of the four CS–orientations. Data and effect sizes are shown as a Cumming estimation plot (http://www.estimationstats.com). Top row, Swarm plots show the z-standardized discrimination indices per frequency (each dot is the discrimination index of one participant). Group statistics are indicated to the right of each swarm as gapped lines (gap = mean, line length = 1 SD). Bottom row, Effect size estimates (Hedges’ g, black dots) for the relevant comparisons (in-phase vs out-of-phase within theta and delta frequency) and their 95% confidence interval (CI; vertical error bars). The unpaired Hedge’s g: for the theta frequency our-of-phase (n = 40) minus Theta in-phase (n = 40): –0.403 [95% CI, -0.855, 0.0389]; for the delta frequency out-of-phase (n = 40) minus in-phase (n = 40): -0.18 [95% CI, –0.619, 0.273]. The 5000 bootstrap samples were taken for CI estimation; the CI is bias corrected and accelerated. (TIF) [file pone.0281644.s006.tif]

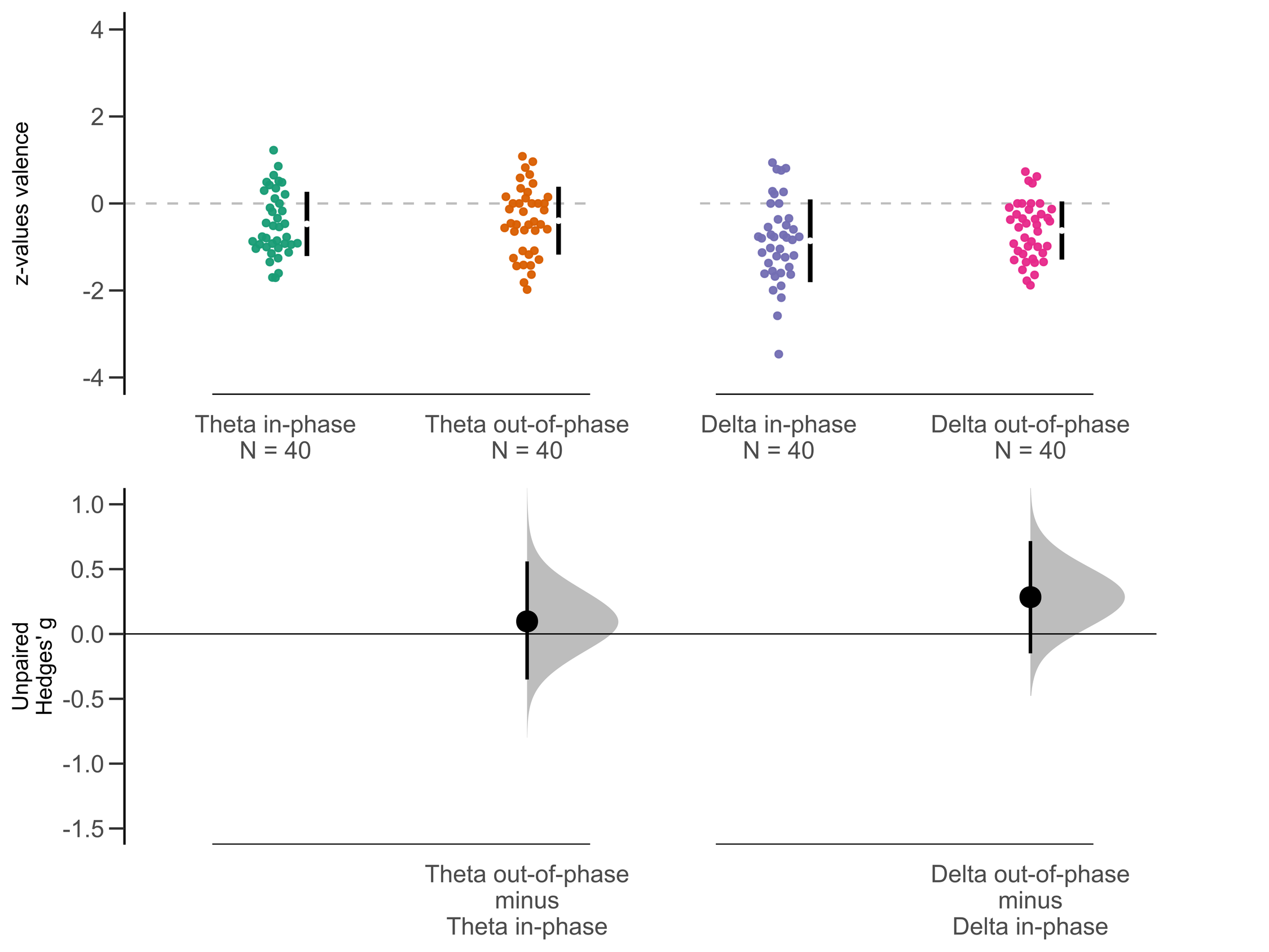

Supplement: S3 Fig — The discrimination index was computed as the difference between the reinforced 45° orientation (CS+) grating and the average of the four CS–orientations. Data and effect sizes are shown as a Cumming estimation plot (http://www.estimationstats.com). Top row, Swarm plots show the z-standardized discrimination indices per frequency (each dot is the discrimination index of one participant). Group statistics are indicated to the right of each swarm as gapped lines (gap = mean, line length = 1 SD). Bottom row, Effect size estimates (Hedges’ g, black dots) for the relevant comparisons (in-phase vs out-of-phase within theta and delta frequency) and their 95% confidence interval (CI; vertical error bars). The unpaired Hedge’s g: for the theta frequency out-of-phase (n = 40) minus theta in-phase (n = 40): 0.11 [95% CI, -0.327, 0.538]; for the delta frequency out-of-phase (n = 40) minus in-phase (n = 40): 0.259 [95% CI, -0.207, 0.665]. The 5000 bootstrap samples were taken for CI estimation; the CI is bias corrected and accelerated. (TIF) [file pone.0281644.s007.tif]

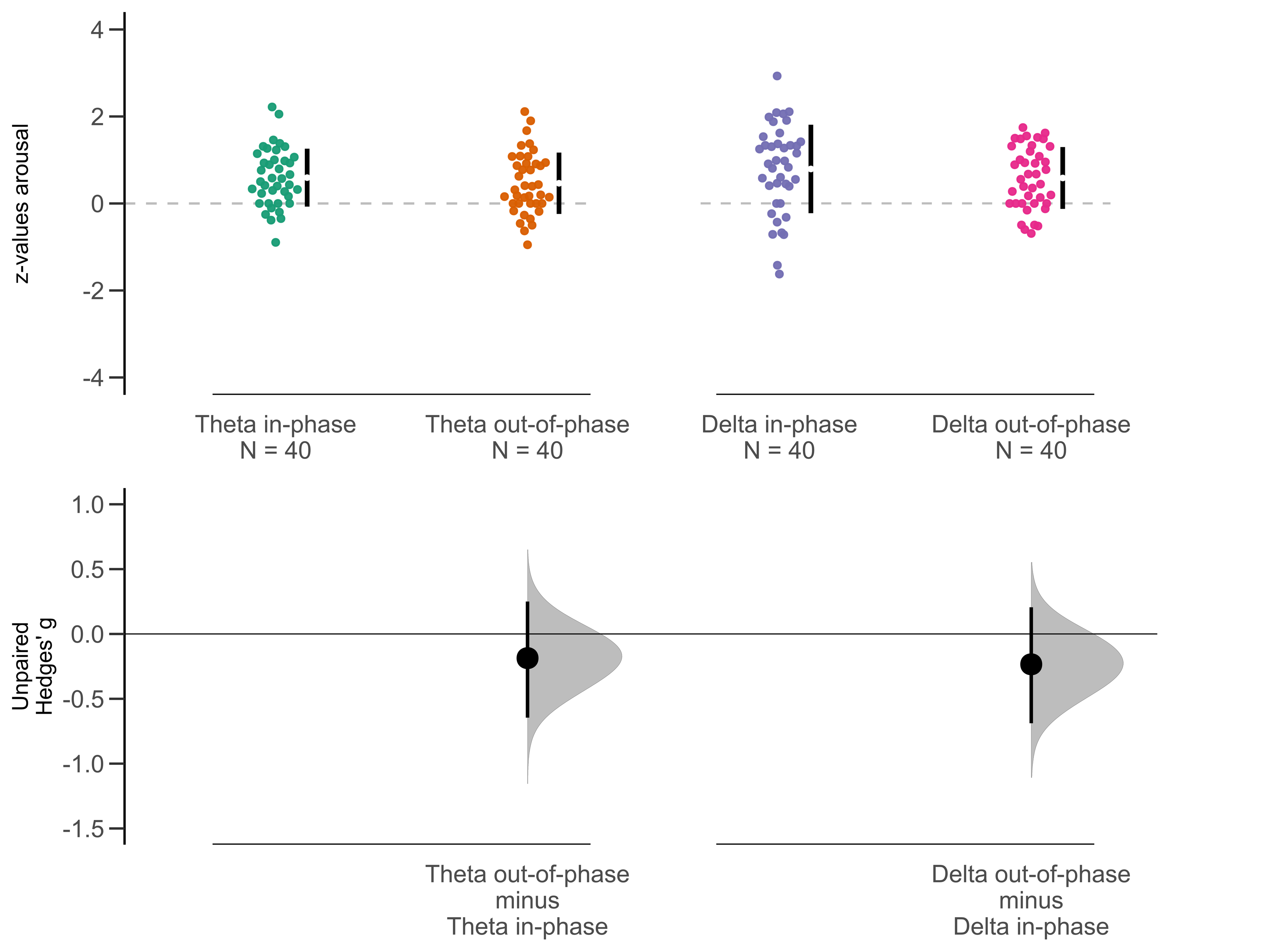

Supplement: S4 Fig — The discrimination index was computed as the difference between the reinforced 45° orientation (CS+) grating and the average of the four CS–orientations. Data and effect sizes are shown as a Cumming estimation plot (http://www.estimationstats.com). Top row, Swarm plots show the z-standardized discrimination indices per frequency (each dot is the discrimination index of one participant). Group statistics are indicated to the right of each swarm as gapped lines (gap = mean, line length = 1 SD). Bottom row, Effect size estimates (Hedges’ g, black dots) for the relevant comparisons (in-phase vs out-of-phase within theta and delta frequency) and their 95% confidence interval (CI; vertical error bars). The unpaired Hedge’s g: for the theta frequency our-of-phase (n = 40) minus theta in-phase (n = 40): -0.204 [95% CI, -0.643, 0.24]; for the delta frequency out-of-phase (n = 40) minus in-phase (n = 40): -0.212 [95% CI, -0.639, 0.239]. The 5000 bootstrap samples were taken for CI estimation; the CI is bias corrected and accelerated. (TIF) [file pone.0281644.s008.tif]

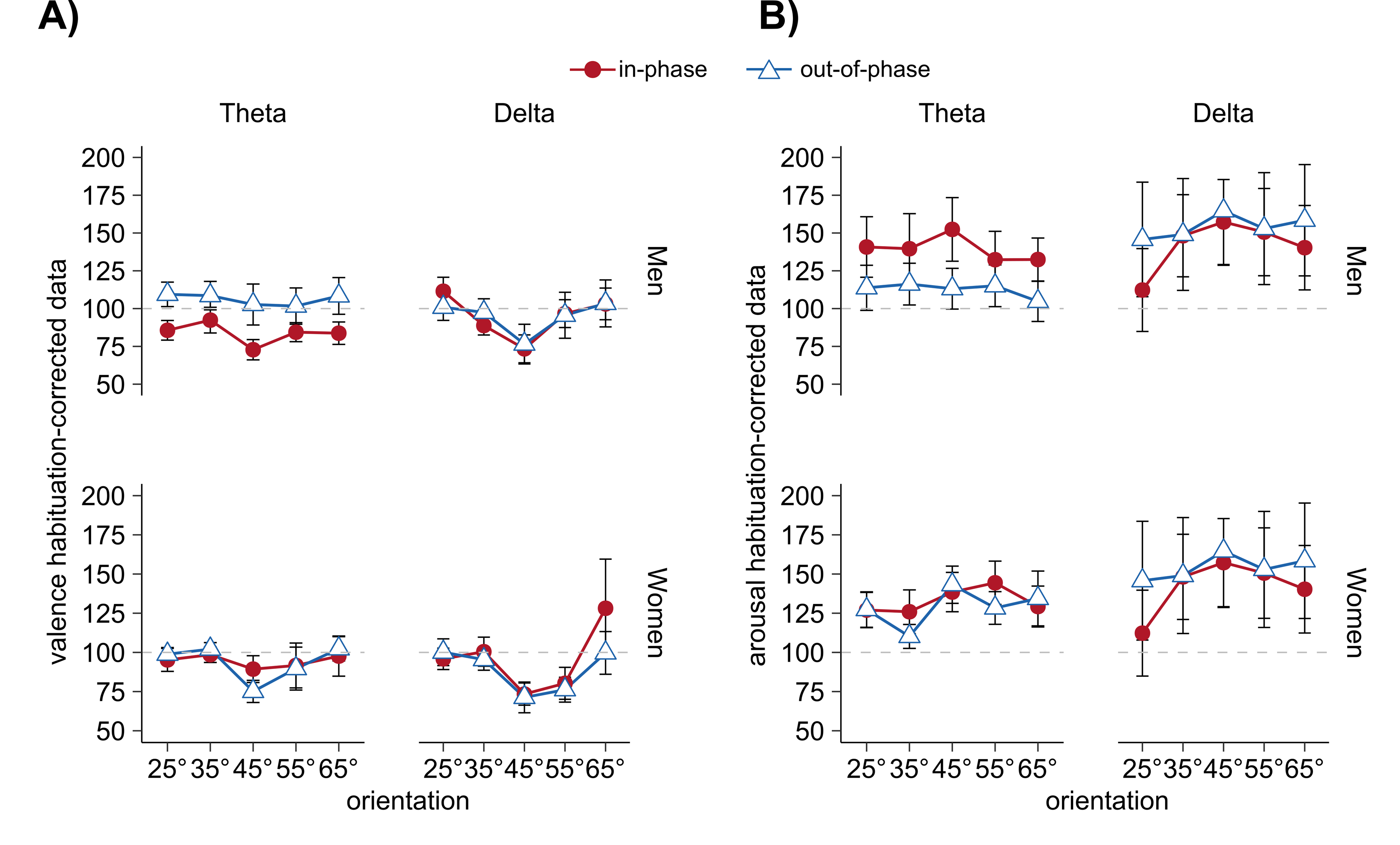

Supplement: S5 Fig — To improve the visualization of the fear generalization pattern for men and women separately, we corrected for the linear trend during habituation, acquisition, and extinction, conducting a habituation correction for valence (A) and arousal (B) data. Both valence and arousal data show ratings after acquisition for each frequency (theta vs. delta) and synchronization (in-phase vs. out-of-phase), separated by sex: within each subplot, top row shows men (n = 80), bottom row shows women (n = 80). Each data point represents mean, habituation-corrected values for each CS orientation, separately for frequency, synchronization, and sex. Error bars show ±1 SEM. (TIF) [file pone.0281644.s009.tif]
